# Systems spatiotemporal dynamics of traumatic brain injury at single-cell resolution reveals humanin as a therapeutic target

**DOI:** 10.1007/s00018-022-04495-9

**Published:** 2022-08-11

**Authors:** Douglas Arneson, Guanglin Zhang, In Sook Ahn, Zhe Ying, Graciel Diamante, Ingrid Cely, Victoria Palafox-Sanchez, Fernando Gomez-Pinilla, Xia Yang

**Affiliations:** 1 Department of Integrative Biology and Physiology, University of California, Los Angeles, Los Angeles, CA 90095 USA; 2 Bioinformatics Interdepartmental Program, University of California, Los Angeles, Los Angeles, CA 90095 USA; 3 Department of Neurosurgery, University of California, Los Angeles, Los Angeles, CA 90095 USA; 4 Brain Injury Research Center, University of California, Los Angeles, Los Angeles, CA 90095 USA; 5 Institute for Quantitative and Computational Biosciences, University of California, Los Angeles, Los Angeles, CA 90095 USA; 6 Molecular Biology Institute, University of California, Los Angeles, Los Angeles, CA 90095 USA; 7 Brain Research Institute, University of California, Los Angeles, Los Angeles, CA 90095 USA

**Keywords:** Traumatic brain injury, TBI, Astrocytes, *Mt-Rnr2*, Humanin, Single-cell RNA sequencing

## Abstract

**Background:**

The etiology of mild traumatic brain injury (mTBI) remains elusive due to the tissue and cellular heterogeneity of the affected brain regions that underlie cognitive impairments and subsequent neurological disorders. This complexity is further exacerbated by disrupted circuits within and between cell populations across brain regions and the periphery, which occur at different timescales and in spatial domains.

**Methods:**

We profiled three tissues (hippocampus, frontal cortex, and blood leukocytes) at the acute (24-h) and subacute (7-day) phases of mTBI at single-cell resolution.

**Results:**

We demonstrated that the coordinated gene expression patterns across cell types were disrupted and re-organized by TBI at different timescales with distinct regional and cellular patterns. Gene expression-based network modeling implied astrocytes as a key regulator of the cell–cell coordination following mTBI in both hippocampus and frontal cortex across timepoints, and *mt-Rnr2*, which encodes the mitochondrial peptide humanin, as a potential target for intervention based on its broad regional and dynamic dysregulation following mTBI. Treatment of a murine mTBI model with humanin reversed cognitive impairment caused by mTBI through the restoration of metabolic pathways within astrocytes.

**Conclusions:**

Our results offer a systems-level understanding of the dynamic and spatial regulation of gene programs by mTBI and pinpoint key target genes, pathways, and cell circuits that are amenable to therapeutics.

**Supplementary Information:**

The online version contains supplementary material available at 10.1007/s00018-022-04495-9.

## Background

Mild traumatic brain injury (mTBI) or concussive injury comprises over 90% of the brain injuries in the United States and can lead to deficits in neuronal function and cognitive abilities that can persist for years after the initial incident [1–3]. Neurons that survive the injury exhibit a decline in function [4, 5], and many patients become vulnerable to a large number of neuropsychiatric and cognitive disorders [2, 3] such as Alzheimer’s disease (AD), posttraumatic stress disorder (PTSD), epilepsy, and anxiety [6–9].

The broad spectrum of clinical symptoms and behavioral manifestations of mTBI reflects a highly complex brain pathophysiology that evolves over time across the complex cytoarchitecture of brain regions. Failure in cognitive processing post-TBI has been associated with dysfunctions of the hippocampus [1, 10] and the cerebral frontal cortex [2]. The hippocampal formation is the main locus for cognitive processing involving learning and memory, which are associated with cognitive disorders such as AD and PTSD. The frontal cortex is critical for problem solving, memory, judgment, and impulse control, and is implicated in the pathophysiology of major depression, PTSD, and schizophrenia. In addition to the central nervous system, the systemic immune system plays an important role in the response to acute and subacute injury and can serve as a clinically relevant source of prognostic and diagnostic biomarkers. In addition to spatial heterogeneity, mTBI also exhibits distinct pathological features at acute, subacute, and chronic stages [11, 12]. To date, the regulatory mechanisms underlying these spatiotemporal changes in mTBI pathology remain unclear, especially at the level of the cell.

We previously used single-cell RNA sequencing (scRNAseq) [13] to dissect the complex pathophysiology underlying mTBI in the heterogenous hippocampus tissue using a mild fluid percussion injury (FPI) mouse model [14]. We were able to prioritize the hippocampal cell types most vulnerable to mTBI at the acute phase. Here, we aim to understand the spatiotemporal dynamics of TBI across two brain regions (hippocampus, frontal cortex) as a function of post-TBI time. In addition, we analyzed individual immune cells in peripheral blood to elucidate systemic immune system response to TBI and to identify peripheral biomarkers.

**Fig. 1:**
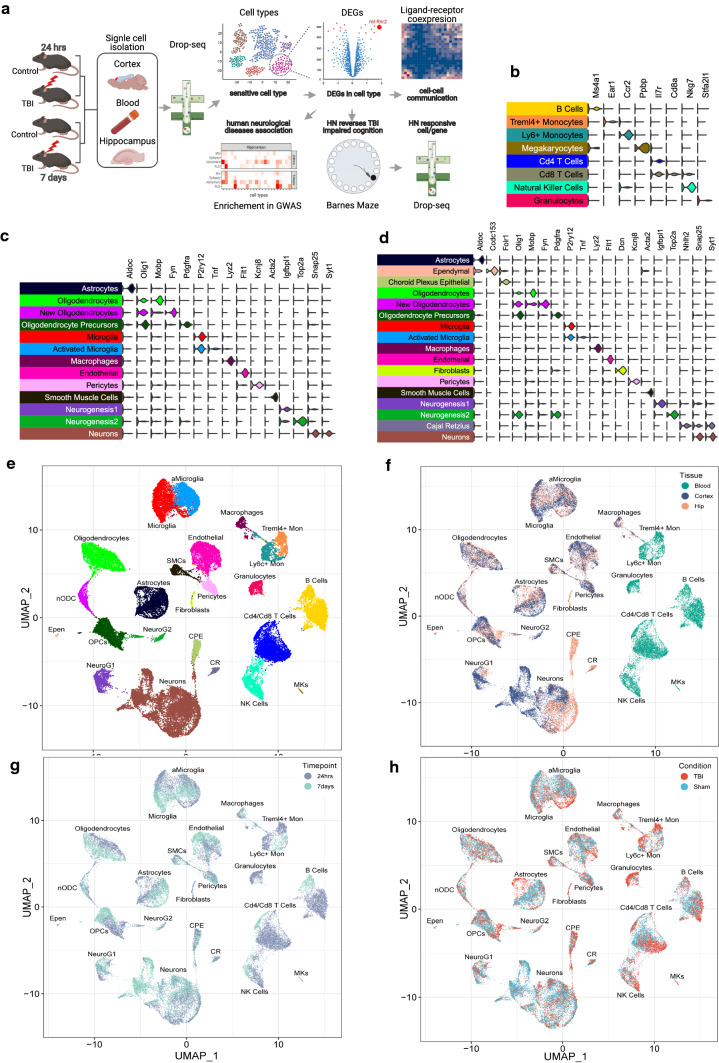
Overall study design and scRNAseq cell clusters and gene markers. **a** Overall study design. **b**–**d** Expression of cell markers for each cell type in peripheral blood (**b**), frontal cortex (**c**), and hippocampus (**d**). **e**–**h** UMAP embeddings of 78,895 cells according to cell types (**e**), tissues (**f**; frontal cortex, hippocampus, and peripheral blood), timepoints (**g**; 24-h vs 7-day), and conditions (**h**; TBI vs sham control). Each point represents a single cell. Cells are clustered based on transcriptome similarity using Louvain clustering and cell types are identified using canonical markers and labeled on the plot. Within each tissue and timepoint, there are *n* = 3 animals per group. HN: Humanin

This study represents the first multi-tissue, multi-timepoint systems-level investigation of the mTBI pathophysiology at single-cell resolution (Fig. 1a). Our findings offer unparalleled insights into the spatiotemporal pathophysiology of mTBI by answering the following longstanding questions, such as: Which cell types, genes, and pathways are most sensitive to mTBI in a spatial- or temporal-specific manner? How do cells relate to each other to coordinate a response to mTBI at different stages in different brain regions? Which cell types in specific brain regions are involved in behaviors associated with psychiatric and neurological disorders? Could the spatiotemporal patterns of single-cell gene regulation guide target prioritization and mTBI therapy? Could the peripheral blood inform on pathology in brain tissues and help identify diagnostic and prognostic biomarkers? Our studies offer critical answers for these questions. To address the therapeutic relevance of the results, we prioritized *mt-Rnr2*, a gene encoding the mitochondrial peptide humanin, as a key target for intervention. Humanin treatment improved cognitive ability following mTBI by restoring metabolic pathways in key cell populations such as astrocytes.

## Results

### Overall study design

As depicted in Fig. 1a, we conducted scRNAseq on the central nervous system (hippocampus and frontal cortex) and circulation (peripheral blood leukocytes) from mice with or without TBI treatment at acute (24-h) and subacute (7-day) phases. Sensitive cell types and differentially expressed genes (DEGs) within each cell type were identified, and cellular communications were derived based on ligand–receptor co-expression analysis. To connect the mouse genes with human diseases, enrichment of human GWAS signals of neurological diseases among cell-type-specific DEGs affected by TBI was assessed. We further prioritized the mitochondrial gene *mt-Rnr2*, encoding humanin, as a broad target of TBI across cell types, tissues, and time points, and tested the potential of humanin to improve TBI cognitive outcome and molecular and cellular pathways.

### Unbiased identification of cell identities across tissues and timepoints

We sequenced a total of 78,895 single cells which passed quality control from blood, hippocampus, and frontal cortex at two time points (Supplementary Table 1). A single-cell digital gene expression matrix was generated using a Snakemake [15] workflow of Drop-seq Tools [13] and dropEst [16]. Cells were projected onto two dimensions with uniform manifold approximation and projection (UMAP) [17] and Louvain [18] clustering was used to define cell clusters (“Materials and methods”). Based on the assessment of sequencing depth per cell-type cluster (Supplementary Fig. 1a, b), overall library sequencing depth (Supplementary Fig. 2), batch effect (Supplementary Fig. 3), and the clustering of individual samples (Supplementary Fig. 4), we saw no evidence of technical or batch contribution to cell clusters.

We used canonical correlation analysis (CCA) [19] (“Materials and methods”) to identify cell-type marker genes which were consistent across the different timepoints or conditions. After each tissue was aligned using CCA, cell cluster identities were determined using previously defined cell-type marker genes (Supplementary Table 2) from literature as well as from single-cell transcriptome references based on hippocampus cells and frontal cortex cells from the DropViz mouse brain atlas [20] (“Materials and methods”). We determined the cell-type identities for all cell clusters of the three tissues using known marker genes for 8 blood leukocytes clusters (Fig. 1b; Supplementary Figs. 5, 6), 13 frontal cortex clusters (Fig. 1c; Supplementary Figs. 7, 8), and 17 hippocampal clusters (Fig. 1d; Supplementary Figs. 9, 10). Further subclustering of neuronal populations in hippocampus and frontal cortex revealed 7 and 13 neuronal subtypes, respectively (Supplementary Figs. 11–14). In addition to canonical marker genes, we also identified additional highly expressed marker genes for each cell type in each tissue using our dataset (Supplementary Table 3) (details in “Materials and methods”). The 24 distinct cell clusters (Fig. 1e) showed observable gene expression differences among the three tissue types (Fig. 1f), between the two time points (Fig. 1g), and between TBI and sham controls (Fig. 1h).

### Quantification of dynamic and regional shifts in cell types in response to mTBI

**Fig. 2:**
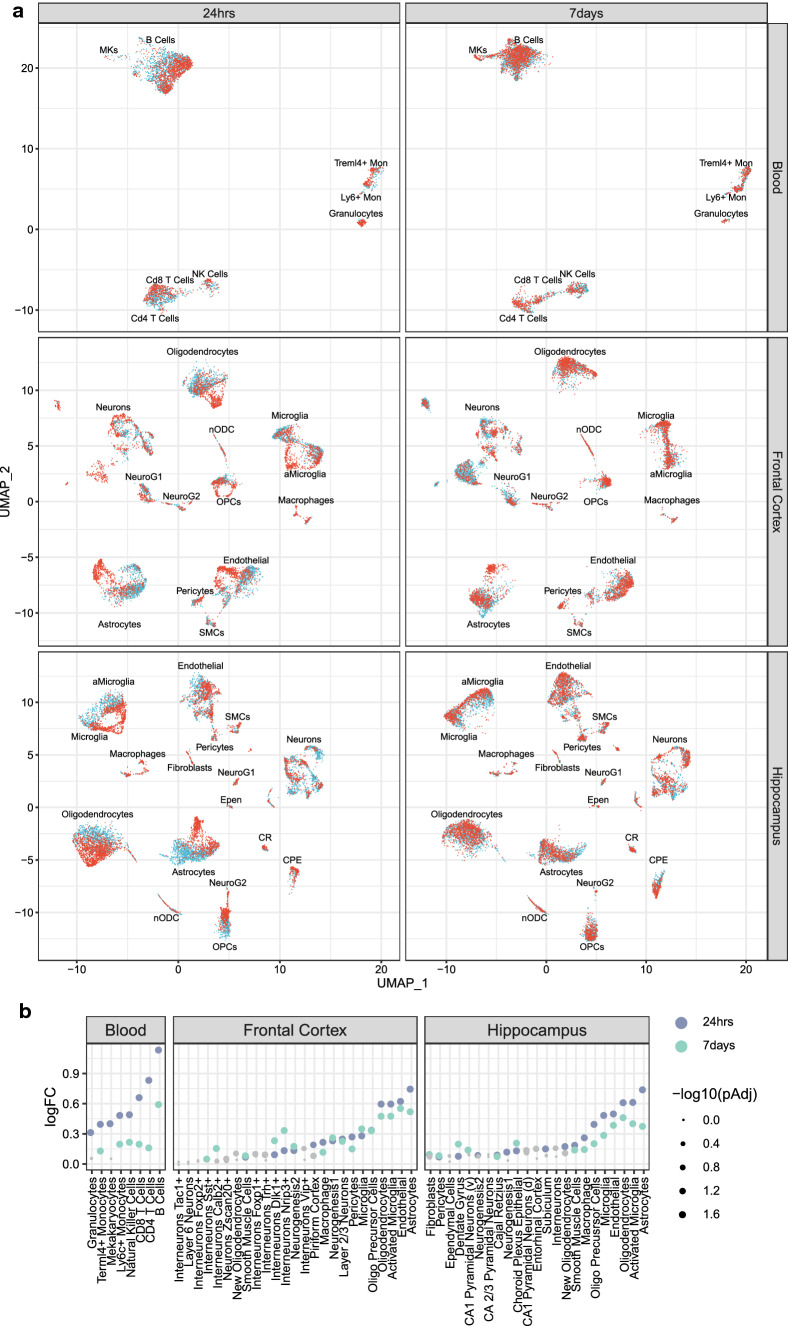
Transcriptomic shifts due to mTBI across cell types in the peripheral blood, frontal cortex, and hippocampus at 24-h and 7-day post-TBI. **a** Difference in the transcriptomes of cells in UMAP for each tissue, timepoint, and mTBI condition, with cells from TBI animals in red and cells from sham control animals in blue. **b** Euclidean distance between TBI and sham control cells within each cell type for each tissue and timepoint. The log fold change (logFC) between the empirical distance and null distribution for each cell type which quantifies the global transcriptome shift is indicated on the *y*-axis. Each point is colored by timepoint and the size of each point relates to the adjusted *p*-value. Gray points do not achieve statistical significance whereas colored dots reach adjusted *p*-value < 0.05

Visual inspection of the UMAP two-dimensional embeddings of single-cell transcriptomes from mTBI and sham control animals revealed striking differences in their gene programs in each tissue at each timepoint (Fig. 2a). To quantify the transcriptomic shifts, we applied three approaches, namely Euclidean distance, subset DEG count, and a support vector machine (SVM)-based classifier to identify top ranked cell types sensitive to mTBI based on the average rank across all three methods with equal weight for each method (Table 1; Supplementary Table 4).

**Table 1:** Consensus ranks of transcriptome perturbation across three different analytical metrics

Tissue	Time	Cell type	ED	SVM	Subset DEG	Avg rank
Blood	24-h	CD8^+^ T cells	3	1	1	1.67
Blood	24-h	CD4^+^ T cells	2	5	1	2.67
Blood	24-h	Ly6c^+^ monocytes	5	3	3	3.67
Blood	7-day	CD8^+^ T cells	4	1	1	2.00
Blood	7-day	Ly6c^+^ monocytes	3	2	3	2.67
Blood	7-day	B cells	1	3	5	3.00
Frontal cortex	24-h	Astrocytes	1	2	1	1.33
Frontal cortex	24-h	Layer 2/3 neurons	8	1	2	3.67
Frontal cortex	24-h	Endothelial	2	6	4	4.00
Frontal cortex	7-day	Endothelial	2	7	5	4.67
Frontal cortex	7-day	Astrocytes	1	8	5	4.67
Frontal cortex	7-day	Activated microglia	3	11	2	5.33
Hippocampus	24-h	Astrocytes	1	1	1	1.00
Hippocampus	24-h	Activated microglia	2	4	2	2.67
Hippocampus	24-h	Microglia	5	5	3	4.33
Hippocampus	7-day	Oligodendrocytes	1	1	5	2.33
Hippocampus	7-day	Astrocytes	4	2	2	2.67
Hippocampus	7-day	Choroid plexus epithelial	6	5	2	4.33

Ranks are calculated within tissue and timepoint. Top 3 cell types are displayed for each tissue and timepoint

ED: Euclidean distance; SVM: support vector machine; DEG: differentially expressed genes

The first method used Euclidean distance to measure the global transcriptomic shift due to mTBI [14], where the rank was determined by the distance in gene expression profiles between TBI and sham control cells for each cell type within each tissue and timepoint with a bigger distance indicating a higher rank (“Materials and methods”). This analysis revealed a general stronger cellular response at 24 h compared to 7 days, particularly in leukocyte populations (Fig. 2b).

The second method quantified the number of statistically significant DEGs between sham control and TBI cells for each cell type using subsampled cells with equivalent number of cells across cell types under the assumption that cell types which are more perturbed by mTBI will have more DEGs. The rank is based on the number of DEGs for each cell type within each tissue and timepoint with a greater number of DEGs resulting in a higher rank. We calculated DEGs on subsampled cell clusters (Supplementary Table 5; Supplementary Fig. 15a, b) to give all cell types equivalent cell number and hence statistical power.

The final method used was a SVM-based classifier, a machine learning-based method [21] (Supplementary Fig. 15c, d), based on the premise that cell types with large differences in their transcriptomes between conditions will achieve high classification accuracy for the conditions. The rank was determined by the median classification accuracy of identifying if a cell came from a TBI or a control sample across 1000 bootstraps for each cell type within each tissue and timepoint.

Given that each method possesses inherent strengths and weaknesses, we considered the consistency across all the methods to rank top sensitive cell types (Table 1; Supplementary Table 4). In peripheral blood, CD8^+^ T cells and Ly6c^+^ monocytes were among the top sensitive cell types in both acute and subacute phases (Table 1). Ly6c^+^ monocytes are known to increase in number following TBI [22] and CD8^+^ T cells are known to infiltrate the brain following injury [23]. Our results point to the timepoints at which these cell types demonstrate major transcriptional shifts. CD4^+^ T cells demonstrated specificity to the 24-h timepoint, which aligns with immunosuppression at the acute phase following mTBI, which specifically affects CD4^+^ T cells [24] (Table 1). B cells demonstrated temporal specificity to the 7-day post-TBI timepoint (Table 1). Notably, B cells are poorly studied with respect to mTBI and may serve as a candidate for future study of the subacute phase.

In the CNS, astrocytes were the top ranked cell type to demonstrate global transcriptional sensitivity in both the hippocampus and frontal cortex at both the acute and subacute phases, highlighting its central role in mTBI (Table 1). Endothelial cells showed transcriptomic alterations across both timepoints specifically in the frontal cortex. The hippocampus experienced a strong immediate immune response with large transcriptomic changes in the microglia and activated microglia in the acute phase. In contrast, the frontal cortex had a more delayed immune response from activated microglia at 7-day post-TBI (Table 1). Within the frontal cortex, layer 2/3 neurons were sensitive at the acute phase of mTBI, agreeing with neuronal hypoexcitation at this timepoint [25]. Oligodendrocytes and choroid plexus epithelial cells both had strong transcriptomic alterations that were specific to the subacute phase in the hippocampus. Both cell types can facilitate the repair process where oligodendrocytes can repair myelin on damaged axons [26] and choroid plexus epithelial cells release growth factors [27] and recruit immune cells [28].

Across tissues and timepoints, our cellular sensitivity analyses based on multiple complementary methods revealed that astrocytes and activated microglia were consistently perturbed across brain regions and timepoints, whereas monocytes, T cells, B cells, neurons, endothelial cells, oligodendrocytes, and choroid plexus epithelia cells were sensitive to mTBI with spatiotemporal specificity and dynamics.

It is important to note that all three methods were designed based on the hypothesis that more sensitive cell types to TBI will demonstrate more transcriptomic changes. In cases where cell sensitivity is reflected by other molecular or cellular features such as protein expression or morphological changes, these methods will miss those cell types.

### mTBI alters cell–cell ligand–receptor co-expression with regional and dynamic specificity

**Fig. 3:**
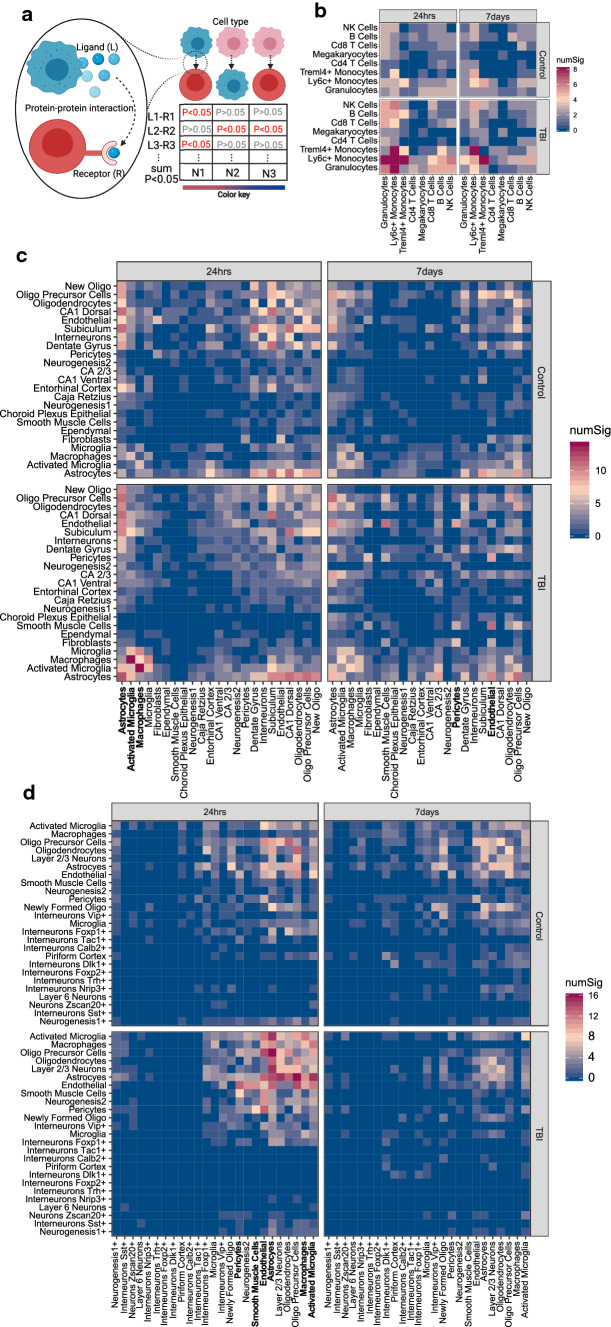
Alterations in ligand–receptor-mediated cell–cell communication in mTBI in individual tissues and timepoints. **a** Schematic diagram of CellPhoneDB which was applied to our single-cell data to infer significant ligand–receptor interactions between pairs of cells within the same tissue. Each plot is split into four panels which denote the timepoint (24-h or 7-day post-TBI) and the condition (sham control or TBI). The rows and columns indicate the interacting cell types determined by the number of ligand–receptor pairs between cell types. The color of each tile denotes the number of significant interactions between the two cell types under the assumption that cell types which are communicating more will have a larger number of ligand–receptor interactions. This method was applied to single-cell data from: **b** peripheral blood, **c** hippocampus, and **d** frontal cortex. The cell types mentioned in the main text were highlighted with red rectangles

To investigate how mTBI influences the coordinated gene expression between cell types, we used the ligand–receptor-based method CellPhoneDB [29] to infer cell–cell gene expression coordination (Fig. 3a). We found a consistent increase in coordinated gene expression patterns across cell types at the acute phase of mTBI across the peripheral blood (Fig. 3b), hippocampus (Fig. 3c), and frontal cortex (Fig. 3d), which mostly subsided at the 7-day phase.

Astrocytes were found at the center of the increased coordinated gene expression patterns across cell types, especially at the acute phase post-TBI in both the hippocampus and frontal cortex (Fig. 3c, d). This is consistent with the high global transcriptional sensitivity of astrocytes across tissues as revealed in the cell sensitivity analysis above (Table 1). There was tight coordination across astrocytes, neuronal populations, and oligodendrocytes at the acute phase post-TBI in both the hippocampus and the frontal cortex. Axonal injury and demyelination are hallmarks of TBI which require oligodendrocytes for repair with signs of myelination known to begin as early as 6 h post-TBI [30]. Additionally, vascular populations, including endothelial cells, pericytes, and smooth muscle cells, were tightly coordinated in the frontal cortex at the acute phase (Fig. 3c, d), whereas the coordination of vascular populations in the hippocampus occurred at the subacute phase, which potentially indicates differential timing of vascular remodeling between brain regions.

Across the brain and periphery, at the acute phase, we observed an increase in coordinated immune cell gene expression patterns across many blood leukocyte cell types (Fig. 3b) as well as between activated microglia and macrophages in the hippocampus (Fig. 3c) and frontal cortex (Fig. 3d). The immune cell coordination was sustained in peripheral blood at the subacute phase (Fig. 3b) but not in the CNS (Fig. 3c, d).

These results uniquely highlight divergent cellular coordination between brain regions and between the CNS and periphery immunity in response to mTBI pathophysiology.

### Dynamic and regional alterations in genes and pathways in mTBI

**Fig. 4:**
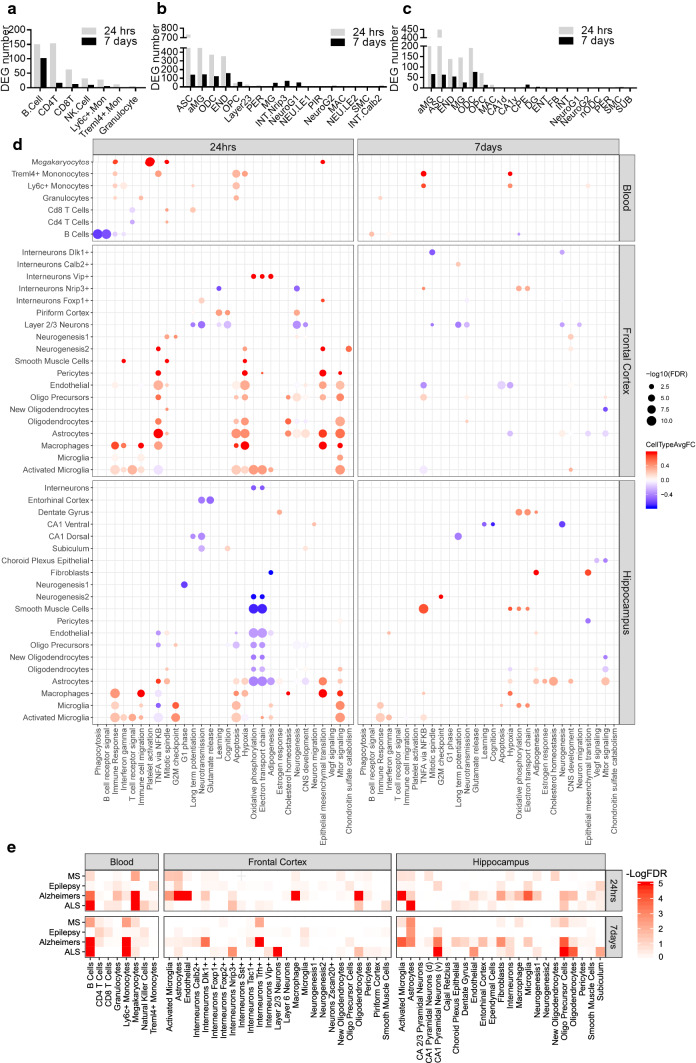
Differentially expressed genes (DEGs) and pathways induced by mTBI across tissues and timepoints and relevance of DEGs to human neurological disorders. **a**–**c** The comparison of DEG number in each cell type induced by TBI between two timepoints for peripheral blood (**a**), frontal cortex (**b**), and hippocampus (**c**). **d** Top enriched pathways induced by mTBI for each tissue and timepoint combination. Each dot is colored by the average log fold change between TBI vs sham control cells within that cell type for significant DEGs which overlap the indicated pathway. The size of each dot is proportional to the −log10(FDR). Cell types and pathways have been clustered with hierarchical clustering. **e** Enrichment of human disease GWAS genes in cell-type DEG gene sets across three tissues and two timepoints as assessed by MSEA in Mergeomics. Color corresponds to −log10(FDR) of the enrichment

To determine the specific genes and pathways that may contribute to mTBI pathogenesis in a regional or dynamic fashion, we identified DEGs in individual cell types (Supplementary Table 6) and annotated them with curated biological pathways (Supplementary Table 7). The immune cells had large numbers of DEGs in the acute phase in peripheral blood (Fig. 4a). In both brain regions, activated microglia, astrocyte, and endothelial cells also had more DEGs in acute phase (Fig. 4b, c). Microglia, Nrip3^+^ interneurons and cells expressing neurogenesis markers had higher number of DEGs at 7-day post-TBI in frontal cortex (Fig. 4b).

The enrichment of numerous pathways among the DEGs in various cell types allowed us to identify both consistent and unique pathways with spatiotemporal specificity (Fig. 4d; Supplementary Table 7). In the acute phase following mTBI, the apoptosis pathway was one of the consistently enriched pathways among DEGs in glial cells across the hippocampus and frontal cortex in addition to several immune cell types in the peripheral blood. The mTOR signaling pathway was also enriched among DEGs in many cell types across the hippocampus and frontal cortex, which is consistent with the role of mTOR signaling in metabolism, growth, proliferation, and survival [31]. There was also an enrichment of immune response pathways in macrophages, microglia, and activated microglia in the hippocampus and frontal cortex as well as in many cell types in the peripheral blood. In the subacute phase, the immune response pathways were no longer enriched in cell types in the peripheral blood and the frontal cortex, but remained enriched in the microglia populations in the hippocampus. Across both the acute and subacute phases, decreased expression of genes from Layer 2/3 neurons in the frontal cortex were enriched for long-term potentiation and neurotransmission.

We also identified pathways that showed both regional and dynamic specificity. Our results showed downregulation of genes involved in oxidative phosphorylation and the electron transport chain across many cell types in the hippocampus in the acute phase, supporting the hippocampus as the main site for the known metabolic suppression of acute mTBI. In the subacute phase, however, there was an increase in oxidative phosphorylation and the electron transport chain gene expression in hippocampal microglia, smooth muscle cells, and dentate gyrus granule cells, demonstrating the dynamic shift in hippocampal cell metabolism between mTBI stages. In the frontal cortex, the hypoxia pathway was enriched in primarily glial cells and vascular cells in the acute phase following mTBI.

The pathways along with their specific cell type, tissue, and injury time context revealed by our analysis portrait the complex and dynamic molecular processes underlying mTBI pathogenesis.

### Enrichment of human neurological disease genes among DEGs altered in cell types following mTBI

To assess the association of the cell-type-specific DEGs for each timepoint and tissue with human diseases, we intersected the DEGs with full summary statistics of human GWAS for 4 neurological diseases, including AD, amyotrophic lateral sclerosis (ALS), epilepsy, and multiple sclerosis (MS), which have been associated with TBI [32–35] (“Materials and methods”). We found significant enrichment of DEGs for GWAS association with neurological diseases, but cellular and gene specificity of disease association differed between tissues and timepoints (Fig. 4e).

Astrocytes have been associated with neurodegenerative diseases [36–38]. Our study provides molecular support for these associations by demonstrating that astrocyte DEGs were strongly enriched for GWAS associations with neurological diseases across timepoints and tissues (Fig. 4e). For instance, in the acute phase, hippocampal astrocyte DEGs showed enrichment for ALS GWAS signals, whereas astrocyte DEGs in the frontal cortex showed enrichment for AD, epilepsy, and MS. At the 7-day timepoint, hippocampal astrocyte DEGs were enriched for genetic signals of MS, AD, and epilepsy.

Ventral CA1 pyramidal neuron DEGs in the hippocampus show an enrichment for ALS and AD GWAS signals in the subacute phase but no enrichment in the acute phase. Likewise, layer 2/3 neuron DEGs in the subacute phase in the frontal cortex had a specific enrichment for ALS associated genetic signals. B-cell DEGs in the peripheral blood had consistent enrichment across timepoints for genetic signals associated with ALS and AD. The role of B cells in AD has been extensively investigated [39, 40], and depletion of B cells showed promise in the reversal of AD progression [41]. However, the association of B cells with ALS has not been established, which could be investigated in the future.

These results suggest that tissue- and injury stage-specific gene alterations in vulnerable cell types to mTBI may contribute to development of neurological diseases and future experimental testing is necessary to confirm disease causality.

### Cell-type-specific DEGs

**Fig. 5:**
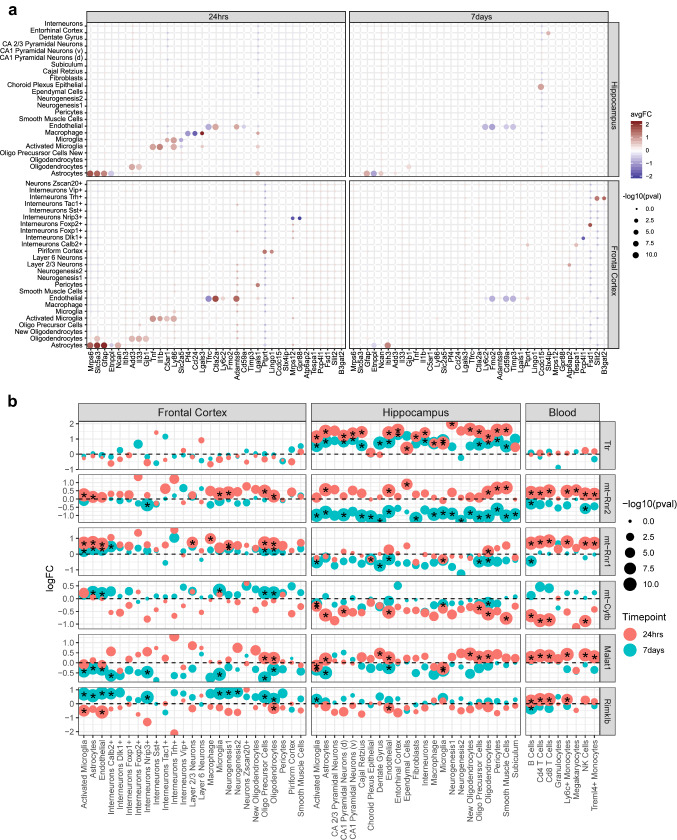
Top cell-type-specific and multi-cell-type DEGs. **a** The top DEGs which were significantly differentially expressed in a single cell type within a particular tissue and timepoint. Each DEG is depicted in a separate column and cell types are indicated by rows. The left panel is from 24-h post-TBI and the right panel is from 7-day post-TBI. The color of each dot indicates the log (fold change) of the bene between TBI and sham control cells (red indicates higher in TBI; cyan indicates lower in TBI) within a particular cell type. The size of each dot corresponds to the −log10(adjusted *p*-value). **b** The top DEGs significantly differentially expressed in the most cell types across tissues and timepoints. Each row depicts a DEG. The genes which are significantly differentially expressed (adjusted *p*-value < 0.05) in specific cell types are indicated by a star. The color of each dot indicates the timepoint (24-h in red and 7-day in blue) at which the DEG was found and the size of the dot corresponds to the −log10(*p*-value). The *y*-axis is the log(fold change) of the gene between TBI and sham control cells within a particular cell type. Cell types are indicated on the *x*-axis

Interrogating cell-type-specific genes perturbed by mTBI can reveal fine dysregulation of microcircuits which can be leveraged for cell-type-specific therapeutic interventions. There are many cell-type-specific DEGs with unique or consistent spatiotemporal specificity (Fig. 5a). Many of these genes have been implicated in the pathophysiology of TBI and related disorders or affect pathways integral to TBI.

We replicated a number of previously reported cell-type-specific DEGs, including *Tnf* and *Il1b* upregulation in microglia [42, 43] and *Ctla2a* and *Adamts9* upregulation in vascular cells in both the frontal cortex and hippocampus in the acute phase [44]. We also found a greater fold change in *Gfap* expression in the frontal cortex compared to the hippocampus in the acute phase as the frontal cortex is closer to the injury location in our experiments, which is consistent with the reported *Gfap* increase relative to severity and proximity to the injury [45]. Increased expression of *Il33* in oligodendrocytes [46] and increased expression of *Ly86* in microglia [47] 24-h post-TBI were also consistent with previous reports and observed in both the frontal cortex and hippocampus.

Novel cell-type-specific DEGs identified from our study include *Etnpll*, which has been previously linked to schizophrenia and bipolar disorder [48] and was found downregulated in astrocytes across both brain regions and timepoints in our study. In the acute phase, downregulation of *Gpr88*, which is implicated in spatial learning and anxiety [49, 50], was specific to Nrip3^+^ interneurons in the frontal cortex; *Tfrc*, which modulates ferroptosis sensitivity [51]—a mechanism of cell death, was downregulated in endothelial cells in the hippocampus and frontal cortex; *Mrps6*, which was specifically upregulated in astrocytes in both the cortex and the hippocampus, has been linked to PD [52]. In the subacute phase, *Timp3*, which aids in neuroprotection [53], was downregulated in endothelial cells in the frontal cortex and hippocampus. *Ncan*, which suppresses axonal regeneration after neural injury [54], was upregulated in astrocytes in the frontal cortex at 24-h post-TBI but at 7-day post-TBI in astrocytes in the hippocampus, thereby suggesting different regional timelines for changes in axonal regeneration.

### Robust mTBI DEGs across spatiotemporal domains

In addition to the above cell-type-specific DEGs, we also identified DEGs altered across cell types, tissues and timepoints. These DEGs may underlie the broad symptomology of mTBI due to their ultra-sensitivity to mTBI across spatiotemporal domains, and may serve as biomarkers that can link mTBI brain pathology with peripheral blood cells.

In our previous study, we identified *Ttr* as a gene which was differentially upregulated in a majority of cell types in the hippocampus at 24 h. This guided our selection of T4 thyroid hormone to test as a protective agent against the cognitive consequences post-TBI [14]. The pan-hippocampal upregulation of *Ttr* post-TBI in the acute phase was confirmed with this independent dataset (Fig. 5b). By expanding the tissues and timepoints in the current study, we were able to reveal that *Ttr* regulation across cell types persisted from 24-h to 7-day post-TBI, which represents a new finding from the current study. The hippocampal specificity of *Ttr*, a main transporter of the T4 thyroid hormone in the brain, is consistent with the regional specificity of the metabolic pathway depression (oxidative phosphorylation and electron transport chain) to the hippocampus (Fig. 4d).

In addition to *Ttr*, we identified numerous additional consistent DEGs across cell types including *Rimklb*, *Malat1*, *mt-Cytb*, *mt-Rnr1*, and *mt-Rnr2* (Fig. 5b). *Rimklb*, which encodes a glutamate ligase, was decreased in cell types in the hippocampus and frontal cortex only in the acute phase. *Rimklb* couples glutamate to the acceptor molecule N-acetylaspartate (NAA) which directly controls the availability of N-acetylaspartyl-glutamate (NAAG) [55, 56], the most prevalent neuroactive peptide in the mammalian CNS. This decrease of *Rimklb*, potentially limiting NAAG, offers molecular support for the suppression of neurotransmission observed across the hippocampus and frontal cortex at the acute phase post-TBI. *Malat1*, which encodes a lncRNA showing potential of neural repair [57, 58], was consistently upregulated across all tissues in the acute phase and, interestingly, downregulated across both brain regions in the subacute phase, demonstrating dynamic temporal specificity. Gene *mt-Cytb*, part of the electron transport chain, was downregulated in the acute and subacute timepoints in the hippocampus. Gene *mt-Rnr2* was upregulated in six or more cell types in each of the tissues profiled in the acute phase post-TBI, but downregulated mainly across the hippocampus cell types in the subacute phase. *mt-Rnr2* encodes the mitochondrial peptide humanin, which has diverse intracellular and extracellular functions and plays an important role in neuroprotection and metabolism [59–69]. These genes represent potential novel targets of the mTBI pathophysiology due to their broad and dynamic alterations across many cell types. Additionally, the consistent expression changes of several of these genes in peripheral blood cells point to the possibility of using these genes as biomarkers of mTBI for the acute (upregulation of *mt-Rnr2*, *mt-Rnr1*, *Malat1*) or subacute phase (downregulation of *mt-Cytb*).

Since multiple mitochondrial genes were among DEGs, we further investigated whether mitochondria content, an indicator of scRNAseq quality, affected the results. All cells passing our quality control had mitochondrial gene content < 15%, and one third of the mitochondrial genes were DEGs in one or more cell types but showed different spatial, temporal, and directional changes (Supplementary Table 6). Therefore, our findings on mitochondrial genes in our DEGs are most likely not due to mitochondria content or scRNAseq quality issues.

### Targeting mt-Rnr2 with humanin treatment reversed cognitive impairment

**Fig. 6:**
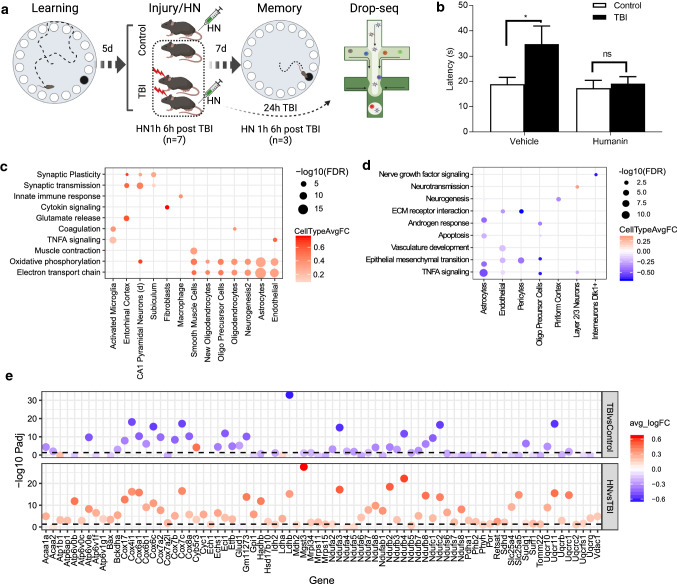
Experimental validation of humanin as a treatment target. **a** Schematic diagram of study design. **b** Bar plot of latency to navigate the maze for sham control and TBI mice treated with vehicle and humanin. Learning was conducted for 4 days prior to injury/surgery and memory was tested 7 days after injury/surgery. Statistics was computed using two-way ANOVA with Bonferroni correction for multiple comparison test. *p < 0.05, ns represents not significant, *n* = 6 per group. **c**, **d** Top enriched pathways of genes reversed by humanin treatment in hippocampus (**c**) and frontal cortex (**d**). Each point is colored by the average log(fold change) between cells from humanin-treated TBI animals and TBI cells within that cell type for significant DEGs which overlap the indicated pathway. The size of each dot corresponds to the −log10(FDR). Cell types and pathways have been clustered with hierarchical clustering. **e** Differentially expressed genes in the oxidative phosphorylation pathway in hippocampal astrocytes at 24-h post-TBI. Genes within the pathway are on the *x*-axis and −log10(adjusted *p*-value) of the differentially expressed gene on the *y*-axis. The color of each dot indicates the fold change between the groups; positive fold change is in red and negative fold change is in blue. The top panel shows differential expression for TBI versus sham control cells and the bottom panel shows differential expression for humanin-treated TBI cells versus TBI cells

To experimentally validate the functional role of the genes demonstrating broad spatiotemporal alterations in mTBI, we focused on *mt-Rnr2*. We postulate that humanin modulates the metabolic crisis in the acute phase and protect from neuronal death in the subacute phase following mTBI. To test this hypothesis, we introduced humanin post-TBI and evaluated cognitive behaviors as determined with a Barnes Maze test, followed by scRNAseq analysis to understand the molecular mechanisms (Fig. 6a). Acute intraperitoneal injection of humanin post-mTBI prevented learning and memory impairment at one-week post-mTBI (Fig. 6b).

To tease apart the underlying mechanisms, we conducted scRNAseq on the frontal cortex and hippocampus of TBI mice with and without humanin treatment (Supplementary Fig. 16). We found that humanin treatment reversed the expression of hundreds of DEGs (Supplementary Table 8) and pathways (Fig. 6c, d; Supplementary Table 9) induced by mTBI across many cell types in both the hippocampus and frontal cortex at 24-h post-mTBI.

In the hippocampus, humanin treatment reversed the metabolic depression (Fig. 6c) observed in astrocytes, oligodendrocyte populations, endothelial cells, and smooth muscle cells under mTBI (Fig. 4d). Astrocytes, known for their role in metabolic support of neurons, showed a strong upregulation in genes in the oxidative phosphorylation pathway after humanin treatment compared to TBI animals (Fig. 6e). Humanin also increased the expression of genes involved in neurotransmission in layer 2/3 supragranular cortical neurons in the frontal cortex as well as in multiple neuronal populations in the hippocampus and mitigated the disruption of the vascular system which can lead to secondary injury in the frontal cortex (Fig. 6c, d).

**Fig. 7:**
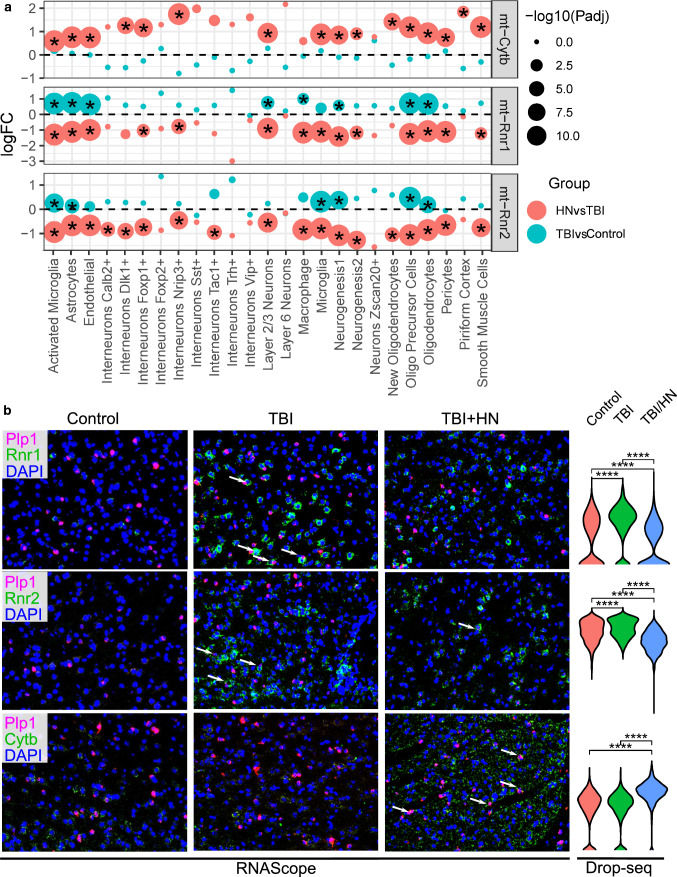
RNAscope validation of select DEGs affected by humanin identified from scRNAseq. **a** Gene expression of *mt-Cytb*, *mt-Rnr1* and *mt-Rnr2* across treatments in different cell populations of cortex with or without humanin (HN). The differentially expressed genes (adjusted *p*-value < 0.05) are indicated by a star. The color of each dot indicates the group which the DEG corresponds to and the size of the dot corresponds to the −log10(adjusted *p*-value). The *y*-axis is the log (fold change) of the gene between TBI and sham control or between TBI/Vehicle and TBI/HN cells within a particular cell type (indicated on the *x*-axis). **b** Validation of gene expression changes of *mt-Cytb*, *mt-Rnr1* and *mt-Rnr2* in response to TBI with or without HN in oligodendrocytes of cortex using RNAscope. *Plp1* was used as oligodendrocytes marker and was stained in pink. The target DEGs *mt-Cytb*, *mt-Rnr1* and *mt-Rnr2* were stained in green. The arrows indicate the overlap between marker gene and target DEGs. The expression of each target DEG determined by scRNAseq is displayed as violin plots and Wilcoxon rank-sum test was used to determine statistical significance between sham control, TBI and TBI/HN groups and adjusted *p*-value was calculated. ****p < 1 × 10^−4^, ns: *p* > 0.05

We also further validated select DEGs in oligodendrocytes of cortex using RNAscope. We found the *mt-Rnr1* and *mt-Rnr2* expression levels were enhanced in response to TBI injury, but were normalized by humanin treatment. The expression of another mitochondrial gene *mt-Cytb*, which showed decreased expression in numerous hippocampal and blood cell types post-TBI, was elevated by humanin treatment (Fig. 7).

Overall, the phenotypic and molecular reversals by humanin treatment support that humanin is a regulator that corrects diverse processes in numerous cell types involved in mTBI.

## Discussion

In this first systems-level investigation of individual cell types across three tissues and two timepoints using high-throughput scRNAseq, we determined differential cellular and molecular sensitivity to mTBI and revealed comprehensive and novel molecular insights into the spatiotemporal gene regulation of mTBI response and their connections to pathophysiological consequences. The specific cell types, pathways, and cell–cell interactions that are affected across different tissues and timepoints with varying spatiotemporal sensitivity provide a comprehensive cellular map for prioritizing the most vulnerable cell types, genes, and pathways for intervention at the right time point, tissue site, and cell types.

In hippocampus, previous studies have mainly noted metabolic depression as a known consequence of TBI [70–73]. Instead, we found cellular and temporal specificity in hippocampus with respect to metabolic regulation. We observed downregulation of genes involved in oxidative phosphorylation and the electron transport chain in astrocytes, oligodendrocytes, vascular cells, and interneurons in the acute phase, but a boost in metabolic pathways in dentate gyrus granule cells, smooth muscle cells, and microglia in the subacute phase. The temporal specificity of metabolic regulation in specific cell types at specific timepoints supports the need for precision targeting of hippocampal cellular metabolism in mTBI.

In the frontal cortex, we observed regional specificity with respect to endothelial cells, which had large alterations in their global transcriptomic profiles in both the acute and subacute phases and demonstrated tight gene to gene transcriptional coordination in the acute phase post-TBI. Injuries to the microvasculature can lead to neurodegenerative disease [74], making this an attractive target for future therapeutic interventions. In addition, the hypoxia pathway also exhibited a regional specificity to the cortex across many cell types in the acute phase following mTBI. It has been previously shown that the frontal lobe is particularly sensitive to hypoxia in the acute phase following mTBI [75, 76], making this an important tissue and timepoint to target for intervention to mitigate hypoxia-related consequences of mTBI. However, it is important to consider that changes in pathways like hypoxia may promote pathology or confer neuroprotection [77–81]. Therefore, it is important to tease apart protective versus deleterious effects for the implicated pathways.

Across brain sites and injury stages, we identify astrocytes as a key cell type to the mTBI pathology. Astrocytes are known to play an important role in the acute and chronic responses to mTBI and the resulting changes in gene expression, morphology, proliferation and function are known as astrogliosis [45, 82, 83]. The diverse functions of astrocytes are central to the mTBI pathogenesis and explain why astrocytes have such massive global transcriptional changes across regions and timepoints. Our cell–cell coordination analysis based on ligand–peptide gene coregulation also provided evidence supporting astrocytes as key regulators of the other cell types and processes. In particular, astrocytes are highly connected to cell types such as neurons, especially in the acute phase in the frontal cortex and the hippocampus. These neuronal populations may be particularly vulnerable to metabolic depression and astrocytes may serve to stabilize or inhibit neuronal circuit function following mTBI.

In the peripheral blood, CD8^+^ T cells and Ly6c^+^ monocytes were found to be sensitive to mTBI in both acute and subacute phases, whereas CD4^+^ T cells showed specificity to the acute phase and B cells showed specificity to the subacute phase. Immune response pathways were also found to be significantly altered in immune cells in the acute phase of TBI. The strong transcriptional perturbations in peripheral immune cells support adaptive immune activation to the CNS injury signals. In contrast to previous reports on the lack of effect of TBI on B-cell biology [84–86], our study found that B cells displayed perturbations at 7-day post-TBI. B cells may play a role in neuronal function recovery post-TBI, as previously observed in stroke induced brain injury [87].

Comparison of DEGs across tissues, cell types, and time points also revealed numerous cell-type-specific and cell-type-independent genes responsive to mTBI. Examples of cell-type-specific DEGs include endothelial-specific *Tfrc* at the acute phase and *Timp3* at the subacute timepoint, and astrocyte-specific *Ncan* in the acute phase in hippocampus and in the subacute phase in frontal cortex. *Tfrc* is known to be highly expressed in brain endothelial cells and responsible for ion homeostasis maintenance [88, 89], while increased *Timp3* expression in cerebral vessels is associated with amyloid angiopathy [53, 90–92]. *Ncan* encodes neurocan, which is produced by astrocytes and may interfere with axonal regeneration after CNS injury [54, 93]. Examples of highly sensitive genes across cell types, tissues, and stages include *mt-Rnr2, Malat1,* and *Rimklb.* These genes serve as promising targets for future mechanistic studies. As a functional validation of *mt-Rnr2*, treatment of mTBI-induced animals with an i.p. injection of humanin protected against learning and memory deficits and reversed numerous mTBI-perturbed pathways including metabolic depression, specifically in astrocytes, which may allow the neurons to maintain normal function and thus protect learning and memory. Interestingly, alteration of *mt-Rnr2* is also consistent in blood leukocyte populations, making it a potential biomarker accessible in the peripheral blood.

mTBI is the most prevalent form of brain injury and is associated with severe downstream neurological consequences. We found evidence supporting that genes and pathways in individual cell types, tissues, and timepoints sensitive to mTBI are enriched for genetic association signals for neurological disorders associated with TBI including AD, MS, epilepsy, and ALS. Pathways such as metabolic depression, neurotransmission, blood vessel disruption and repair, and immune response and their specific cellular, tissue, and injury stage context likely underline the disease associations.

## Conclusions

In summary, our single-cell resolution study across tissues and timepoints offers the first spatiotemporal cellular and molecular atlas of mTBI using a rodent model. The novel genes and pathways along with their cell type, tissue, and timepoint specificity warrant future functional studies to investigate their causal role in TBI pathology or protection. Our findings serve as the basis to prioritize cell types and gene targets for intervention to mitigate the broad downstream neurological consequences associated with TBI. We acknowledge that the rodent FPI model used represents a specific type of mTBI and future investigation of other injury models is warranted. We also acknowledge that it is critical to study both sexes. Due to the high cost of scRNAseq and the extensive coverage of tissues, timepoints, and biological replicates, we conducted our studies in males here and will examine females in future studies.

## Materials and methods

### Animals and mild fluid percussion injury (FPI)

Ten-week-old male C57BL/6J (B6) mice (Jackson Laboratory, Bar Harbor, ME, USA) weighing between 20 and 25 g were housed in cages (*n* = 3–4/group) and maintained in environmentally controlled rooms (22–24 °C) with a 12 h light/dark cycle. Mice were randomized to receive either FPI or sham control surgeries, with no investigator blinding. Mice were anesthetized with 3% inhaled isoflurane in an induction chamber and transferred to a nose cone connecting with 1.6% isoflurane for maintenance on a heating pad. FPI was performed with the aid of a microscope [94] (Wild, Heerburg, Switzerland), where a 1.5 mm radius craniotomy was made 2.5 mm posterior to the bregma and 2.0 mm lateral (left) of the midline with a high-speed drill (Dremel, Racine, WI, USA). A plastic injury cap was placed over the craniotomy with silicone adhesive and dental cement. When the dental cement hardened, the cap was filled with 0.9% saline solution. Anesthesia was discontinued, and the injury cap was attached to the fluid percussion device. At the first sign of hind-limb withdrawal to a paw pinch, a mild fluid percussion pulse (1.4–1.6 atm) was administered. We corroborated that the animals receive stable pressure, and we observed the apnea and the recovery of breath after the percussion. We also monitored the time from the impact until the animals wake up and spontaneously right from the supine position to the prone position which falls into the range of 4–8 min. Sham control animals underwent an identical preparation with the exception of the lesion. Immediately following response to a paw pinch, anesthesia was restored and the skull was sutured. Neomycin was applied on the suture and the mice were placed in a heated recovery chamber for approximately an hour before being returned to their cages. After 24 h or 7d, mice were sacrificed and fresh hippocampal and frontal cortex tissue was dissected for use in Drop-seq (*n* = 3/group with one animal per group per day; sample size was determined based on previous single-cell studies that demonstrated sufficient statistical power). All experiments were performed in accordance with the United States National Institutes of Health Guide for the Care and Use of Laboratory Animals and were approved by the University of California at Los Angeles Chancellor’s Animal Research Committee.

### Tissue dissociation for Drop-seq

The protocol by Brewer et al. [95] was used to suspend cells at a final concentration of 100 cells/μl in 0.01% BSA-PBS by digesting freshly dissected hippocampus and frontal cortex tissue with papain (Worthington, Lakewood, NJ, USA). Briefly, hippocampi from the ipsilateral side of the brain and frontal cortices were rapidly dissected on ice. The hippocampi and cortices were transferred into 4 ml HABG (Fisher Scientific, Hampton, NH, USA) and incubated in water bath at 30 °C for 8 min. The supernatant was discarded and the remaining tissue was incubated with papain (12 mg in 6 ml HA-Ca) at 30 °C for 30 min. After incubation, the papain solution was removed from the tissue and washed with HABG three times. Using a siliconized 9-in Pasteur pipette with a fire-polished tip, the solution was triturated approximately ten times in 45 s. Next, the cell suspension was carefully applied to the top of the prepared OptiPrep density gradient (Sigma Aldrich, St. Louis, MO, USA) and floated on top of the gradient. The gradient was then centrifuged at 800*g* for 15 min at 22 °C. We aspirated the top 6 ml containing cellular debris. To dilute the gradient material, we mixed the desired cell fractions with 5 ml HABG. The cell suspension containing the desired cell fractions was centrifuged for 3 min at 22 °C at 200*g*, and the supernatant containing the debris was discarded. Finally, the cell pellet was loosened by flicking the tube and the cells were re-suspended in 1 ml 0.01% BSA (in PBS). This final cell suspension solution was passed through a 40-micron strainer (Fisher Scientific, Hampton, NH, USA) to discard debris, followed by cell counting. Separation of choroid plexus from hippocampus is particularly challenging due to the proximity of these two regions. Hence, the hippocampus we dissected included choroid plexus.

### Peripheral blood preparation for Drop-seq

Retroorbital blood was collected in EDTA-treated collection tubes (BD Microtainer MAP Microtube, NJ, USA). Then the blood was mixed with ACK Lysing Buffer (Gibco, NY, USA) at the ratio of 1:20 for 3–5 min at room temperature to lyse red blood cells (RBCs). Leukocytes were separated by centrifugation at 300*g* for 5 min at room temperature. The supernatant was removed without touching the pellet. The pellet was re-suspended with cold PBS and centrifuged at 300*g* for 5 min at 4 °C. The final pellet was re-suspended in 0.01% BSA in PBS and filtered by a 40-micron strainer (Fisher Scientific, Hampton, NH, USA).

### Drop-seq single-cell barcoding and library preparation

Barcoded single cells, or STAMPs (single-cell transcriptomes attached to microparticles), and cDNA libraries were generated following the drop seq protocol from Macosko et al. [13]. and version 3.1 of the online Drop-seq protocol (http://mccarrolllab.com/download/905/). Briefly, single-cell suspensions at 100 cells/μl, EvaGreen droplet generation oil (Bio-Rad, Hercules, CA, USA), and ChemGenes barcoded microparticles (ChemGenes, Wilmington, MA, USA) were co-flowed through a FlowJEM aquapel-treated Drop-seq microfluidic device (FlowJEM, Toronto, Canada) at recommended flow speeds (oil: 15,000 μl/h, cells: 4000 μl/h, and beads 4000 μl/h) to generate STAMPs. The following modifications were made to the online published protocol to obtain enough cDNA as quantified by a high sensitivity BioAnalyzer (Agilent, Santa Clara, CA, USA) to continue the protocol: (1) The number of beads in a single PCR tube was 4000. (2) The number of PCR cycles was 4 + 11 cycles. (3) Multiple PCR tubes were pooled. The libraries were then checked on a BioAnalyzer high sensitivity chip (Agilent, Santa Clara, CA, USA) for library quality, average size, and concentration estimation. The samples were then tagmented using the Nextera DNA Library Preparation kit (Illumina, San Diego, CA, USA) and multiplex indices were added. After another round of PCR, the samples were checked on a BioAnalyzer high sensitivity chip for library quality check before sequencing. A cell doublet rate of 5.6% was obtained by running the microfluidic device without the lysis buffer and counting the percentage of cell doublets through three separate runs.

### Illumina high-throughput sequencing of Drop-seq libraries

The Drop-seq library molar concentration was quantified by Qubit Fluorometric Quantitation (ThermoFisher, Canoga Park, CA, USA) and library fragment length was estimated using a Bioanalyzer. Sequencing was performed on an Illumina HiSeq 4000 (Illumina, San Diego, CA, USA) instrument using the Drop-seq custom read 1B primer (GCCTGTCCGCGGAAGCAGTGGTATCAACGCAGAGTAC) (IDT, Coralville, IA, USA) and PE100 reads were generated. Read 1 consists of the 12 bp cell barcode, followed by the 8 bp UMI, and the last 80 bp on the read are not used. Read 2 contains the single-cell transcripts.

### Drop-seq data pre-processing and quality control

Demultiplexed fastq files generated from Drop-seq were processed to digital expression gene matrices (DGEs) using Drop-seq tools version 1.13 (https://github.com/broadinstitute/Drop-seq) and dropEst [16]. The workflow is available as modified version of the snakemake-based dropSeqPipe (https://github.com/Hoohm/dropSeqPipe) workflow and is available on github (https://github.com/darneson/dropSeqPipeDropEST). Briefly, fastq files were converted to BAM format and cell and molecular barcodes were tagged. Reads corresponding to low quality barcodes were removed and any occurrence of the SMART adapter sequence or polyA tails found in the reads was trimmed. These cleaned reads were converted back to fastq format to be aligned to the mouse reference genome mm10 using STAR-2.5.0c. After the reads were aligned, the reads which overlapped with exons, introns, and intergenic regions were tagged using a RefFlat annotation file of mm10. To make use of reads aligning to intronic regions, which are not considered in Drop-seq tools v1.13, we used dropEst to construct digital gene expression matrices from the tagged, aligned reads where each row in the matrix is the read count of a gene and each column is a unique single cell. The count values for each cell were normalized by the total number of UMIs in that cell and then multiplied by 10,000 and log transformed. Single cells were identified from background ambient mRNA using thresholds of at least 200 genes and a maximum mitochondrial fraction of 15%.

### Identification of cell clusters

The Seurat R package version 3.0.2 (https://github.com/satijalab/seurat) was used to project all sequenced cells onto two dimensions using UMAP [17] and Louvain [18] clustering was used to assign clusters. For consistent identification of cell types across different conditions (TBI vs sham control) and timepoints (24-h vs 7-day), samples were aligned using CCA [96] at the group level (timepoint + condition within a particular tissue). Specifically, the top 3000 features for each group were identified using variance stabilizing transformation and these were used to identify the top 40 CCs across the groups which were then used to find integration anchors to align the datasets. The integrated data were only used to identify and define cell types, all plots which are not explicitly designated as CCA and all downstream analyses were done on non-integrated data to retain the biological effect of timepoint and condition which is inherently removed during integration. Visualization of non-integrated data was achieved with UMAP and Louvain clustering. The optimal number of PCs used for UMAP and Louvain was determined using the Jackstraw permutation approach and a grid search of the parameters. Similarly, the density used to assign clusters was identified using a parameter grid search.

### Identification of marker genes of individual cell clusters

We defined cell cluster-specific marker genes from our Drop-seq dataset using the FindConservedMarkers function in Seurat across all the samples. Briefly, a Wilcoxon Rank Sum Test is run within each set of samples from a particular timepoint and condition and a meta *p*-value across all timepoints and conditions is computed to assess the significance of each gene as a marker for a cluster. Within each sample, the cells are split into two groups: single cells from the cell type of interest and all other single cells. To be considered in the analysis, the gene had to be expressed in at least 10% of the single cells form one of the groups and there had to be at least a 0.25 log fold change in gene expression between the groups. This process was conducted within each sample separately, and then a meta *p*-value was assessed from the *p*-values across all samples. Multiple testing was corrected using the Bonferroni method on the meta *p*-values and genes with an adjusted *p*-value < 0.05 were defined as cell-type-specific marker genes.

### Resolving cell identities of the cell clusters

We used two methods to resolve the identities of the cell clusters. First, we used known cell-type-specific markers curated from literature, single-cell atlases [20, 97–99], previous studies in the hippocampus [100, 101], frontal cortex [102], and blood [103] to find distinct expression patterns in the cell clusters. A cluster showing unique expression of a known marker gene can be used to identify that cell type. We also used a classification approach leveraging the similarities between whole transcriptomes of our data publicly available, large annotated single cell datasets obtained from the DropVIZ mouse brain atlas [20]. Specifically, we obtained the single-cell profiles of 113,171 hippocampus cells and 156,167 frontal cortex cells and their curated cell-type labels from DropVIZ. Using the TransferData function in Seurat, we projected the PCA structure of the relevant reference datasets onto our query single-cell data to classify our single cells as the most likely cell type from the annotated DropVIZ data.

### Confirming absence of batch effect

To quantify the amount of batch effect present between samples within the same group (timepoint + condition) in our dataset, we leveraged the k-nearest-neighbor batch-effect test (kBET) [104]. kBET interrogates the batch labels in local neighborhoods of the single cells and determines if the proportions of the batch labels in these neighborhoods differ from the global distribution. Specifically, a k-nearest neighbor matrix is constructed and 10% of the cells are selected to check the label distribution in that neighborhood. If the label distribution in the local neighborhood is similar to the global label distribution the chi-squared test does not reject the null and the batches are considered well mixed. kBET reports the average test rejection rate, however, we used the acceptance rate which is 1–the rejection rate. In their paper, Büttner et al. noticed that kBET produced lower acceptance rates when used across an entire dataset compared to considering each cell type individually due to variations in cell-type frequencies between samples [104], thus we applied kBET to each cell type separately. To consider a cell type for testing between two samples, we required the presence of at least 15 single cells between the two samples. For each cell type, we ran kBET 100 times and considered acceptance rates of > 0.75 to be indicative of well-mixed batches based on the observed acceptance rates of ~ 0.75–0.9 for each cell type in an experiment in the kBET paper where PBMCs from eight individuals were processed in three batches and demultiplexed with demuxlet [105].

### Quantitative assessment of global transcriptome shifts: Euclidean distance

For each cell type, we generate two representative cells, one for the sham control group and the other for mTBI condition by calculating the average gene expression of each gene for each group within that cell type. We then calculate the Euclidean distance in gene expression between these representative cells as a metric to quantify the effect of TBI on each cell type. We found that the top 1–20 highly expressed genes contributed the vast majority of the signal to this metric when considering normalized expression values. To give genes more equal weight, we transformed the expression of each gene to a z-score for each cell in the given cell type. To circumvent noise arising from lowly expressed genes, we only considered the top 1000 most highly expressed genes in each cell type. To determine if the observed Euclidean distance between sham control and mTBI cells within each cell type is significantly larger than that of random cells, we estimated a null distribution by calculating the Euclidean distance between randomly sampled cells of the given cell type. This permutation approach is repeated for a total of 1000 times to generate the null distribution, which is compared to the Euclidean distance generated from the true TBI and sham control groups to determine an empirical *p*-value. To correct for multiple testing across all the cell types tested, we applied a Bonferroni correction to retrieve adjusted *p*-values.

### Quantitative assessment of global transcriptome shifts: machine learning classifier

For each cell type, a SVM classifier was trained using the ‘caret’ library to predict sham control and TBI labels using the top 1000 most highly expressed genes for that cell type, given there were at least 10 single cells per group. The model was trained 1000 times randomly sampling 70% of the data to train on with tenfold cross validation and 3 repeats and tested on the other 30% of the data which was not seen during the training. The resulting classification accuracies at correctly predicting sham control and TBI labels for held out cells across 1000 permutations were used to generate the box plots.

### Quantitative assessment of global transcriptome shifts: subsample cells

For each cell type, if there were more than 100 cells per condition then they were randomly subsampled to 100 cells. DEGs were then calculated using a Wilcoxon Rank Sum Test (see below) across 1000 permutations and a meta *p*-value was derived using ‘minimump’ from the ‘metap’ package on the Bonferroni corrected *p*-values to get a stable estimate of the number of significant DEGs for each cell type.

### Ligand–receptor cell–cell communication

To infer cell–cell communication, we used the CellPhoneDB [29] ligand–receptor-based method. CellPhoneDB has curated 2486 interactions in the categories of protein–protein interactions, secreted and membrane proteins, and protein complexes. Based on their curated repository, CellPhoneDB predicts enriched receptor–ligand interactions between two cell types based on the expression of a receptor by one cell type and the corresponding ligand by another cell type. Only receptors and ligands which are expressed above 10% in cell-type clusters are considered. To obtain a *p*-value for the interaction a null distribution is obtained by permuting the cluster labels of all cells and comparing the mean expression of the ligand and receptor from the cell types to the null distribution.

### Identification of DEGs between sham control and TBI

Within each identified cell type, sham control and TBI samples are compared for differential gene expression using a Wilcoxon Rank Sum Test. To be considered in the analysis, the gene had to be expressed in at least 10% of the single cells from one of the two groups for that cell type and there had to be at least a 0.25 log fold change in gene expression between the groups. We corrected for multiple testing using Bonferroni correction and genes with an adjusted *p*-value < 0.05 were used in downstream pathway enrichment analyses (unless explicitly noted that a *p*-value of 0.01 was used instead to retrieve suggestive pathways). Enrichment of pathways from KEGG, Reactome, BIOCARTA, GO Molecular Functions, and GO Biological Processes was assessed with Fisher’s exact test, followed by multiple testing correction with the Benjamini–Hochberg method.

### Association of DEGs with human GWAS genes of neurological disorders

Full human GWAS summary statistics of four neurological diseases (MS, epilepsy, Alzheimer’s and PD) were downloaded from the human GWAS catalog on 8-30-2019. To detect the association of cell-type DEGs from TBI with human GWAS genes we used MSEA in the Mergeomics package [106]. Briefly, each GWAS set was first trimmed to remove highly correlated SNPs using the Marker Dependency Filtering function with a LD50 threshold determined using the Hapmap linkage disequilibrium file for the CEU population. For each GWAS, SNPs were mapped to genes using relevant (hippocampus, frontal cortex, and blood) tissue-specific eQTL files from GTEx v8. Once mapped to genes, disease association *p-*values for the corresponding marker were tested for enrichment in the cell-type DEGs gene sets with a chi-squared-like test statistic followed by FDR estimation.

### Humanin treatment

[Gly^14^]-Humanin (humanin, Sigma Chemical Co., St. Louis, MO, USA) dissolved in saline vehicle (154 mM NaCl) was injected i.p. twice at 1 and 6 h after FPI in the treatment group (*n* = 6 mice) at 40 µg/1 kg body weight. 1 µg/100 µl HNG was injected for the mice of average weight of 25 g. Control FPI mice (*n* = 6) received vehicle (saline). The regimen was determined based on a previous study of treatment of ischemia with humanin [107].

### Behavioral tests for humanin treatment experiments

Mice from the sham control, TBI, and humanin treatment groups were trained on the Barnes maze 4 days prior to injury to facilitate learning and tested 7 days after injury to assess memory retention. For learning, animals were trained with two trials per day for 4 consecutive days, and memory retention was assessed 7 days after the last learning trial. The maze was manufactured from acrylic plastic to form a disk 1.5 cm thick and 120 cm in diameter, with 40 evenly spaced 5 cm holes at its edges. The disk was brightly illuminated (900 lumens) by four overhead halogen lamps to provide an aversive stimulus to search for a dark escape chamber hidden underneath a hole positioned around the perimeter of a disk. All trials were recorded simultaneously by a video camera installed directly overhead at the center of the maze. A trial was started by placing the animal in the center of the maze covered under a cylindrical start chamber; after a 10 s delay, the start chamber was raised. A training session ended after the animal had entered the escape chamber or when a pre-determined time (5 min) had elapsed, whichever came first. All surfaces were routinely cleaned before and after each trial to eliminate possible olfactory cues from preceding animals.

### Identification of genes and pathways reversed by humanin treatment

To identify genes reversed by humanin treatment, DEGs were identified for cell type between humanin-treated animals and TBI animals using a Wilcoxon Rank Sum test (as described above). Genes that were significantly differentially expressed between humanin vs TBI samples and TBI vs sham control samples with opposite fold change direction were considered to be reversed by the humanin treatment. Pathway enrichment analysis was conducted on these cell-type DEG gene sets (as described above) to identify pathways reversed by humanin treatment.

### Validation of gene expression using RNAscope

RNAscope Multiplex in situ hybridization (Advanced Cell Diagnostics, Newark, CA, USA) was conducted to evaluate the gene expression as described before [14]. Briefly, the 10 µm brain section was mounted onto gelatin-coated histological slides. The slides were fixed in pre-chilled 4% PFA for 15 min at 4 °C. The section was dehydrated in a series of ethanol followed by treatment of hydrogen peroxide for 10 min and protease IV for 30 min. The probes for the target gene and cell marker gene were mixed and applied to the slides with a 2-h incubation at 40 °C. The slides were incubated with preamplifiers, amplifiers, and dyes specific to probe channel. Finally, the sections were counterstained with DAPI and mounted with ProLong Gold Antifade mountant (Invitrogen, Carlsbad, CA, USA). The following probes were used: Mm-mt-Cytb (Cat. No. 517301); Mm-mt-Rnr1 (Cat. No. 834661); Mm-mt-Rnr2 (Cat. No. 590781); Mm-Plp1-C2 (Cat. No. 428181-C2).

### Supplementary Information

Below is the link to the electronic supplementary material.

Supplementary file 1 (DOCX 5375 KB)

Supplementary file 2 (XLSX 10 KB)

Supplementary file 3 (XLSX 10 KB)

Supplementary file 4 (XLSX 93 KB)

Supplementary file 5 (XLSX 15 KB)

Supplementary file 6 (XLSX 18 KB)

Supplementary file 7 (XLSX 2222 KB)

Supplementary file 8 (XLSX 996 KB)

Supplementary file 9 (XLSX 11 KB)

Supplementary file 10 (XLSX 65 KB)

## Data Availability

The NCBI GEO accession number for the Drop-seq data reported in this paper (fastq files and digital gene expression matrices) is GSE180862.

## Abbreviations

AD: Alzheimer’s disease
ALS: Amyotrophic lateral sclerosis
CCA: Canonical correlation analysis
DGEs: Digital expression gene matrices
FPI: Fluid percussion injury
HN: Humanin
kBET: K-nearest-neighbor batch-effect test
MS: Multiple sclerosis
mTBI: Mild traumatic brain injury
NAA: N-acetylaspartate
NAAG: N-acetylaspartyl-glutamate
PTSD: Posttraumatic stress disorder
RBCs: Red blood cells
scRNAseq: Single-cell RNA sequencing
STAMPs: Single-cell transcriptomes attached to microparticles
UMAP: Uniform manifold approximation and projection

## Code availability

The full code to recreate all analyses and figures in this manuscript is open source and available in R markdown and SnakeMake at https://github.com/darneson/DropSeq.
