# Supplementary material for: Systems spatiotemporal dynamics of traumatic brain injury at single-cell resolution reveals humanin as a therapeutic target
Source: Cell Mol Life Sci. 2022 Aug 11;79(9):480. doi: 10.1007/s00018-022-04495-9 (PMC9372016; doi:10.1007/s00018-022-04495-9)
Supplement: Supplementary file 1 — (DOCX 5375 KB) [file 18_2022_4495_MOESM1_ESM.docx]

**Supplementary Figures**

**Supplementary Figure 1**

**
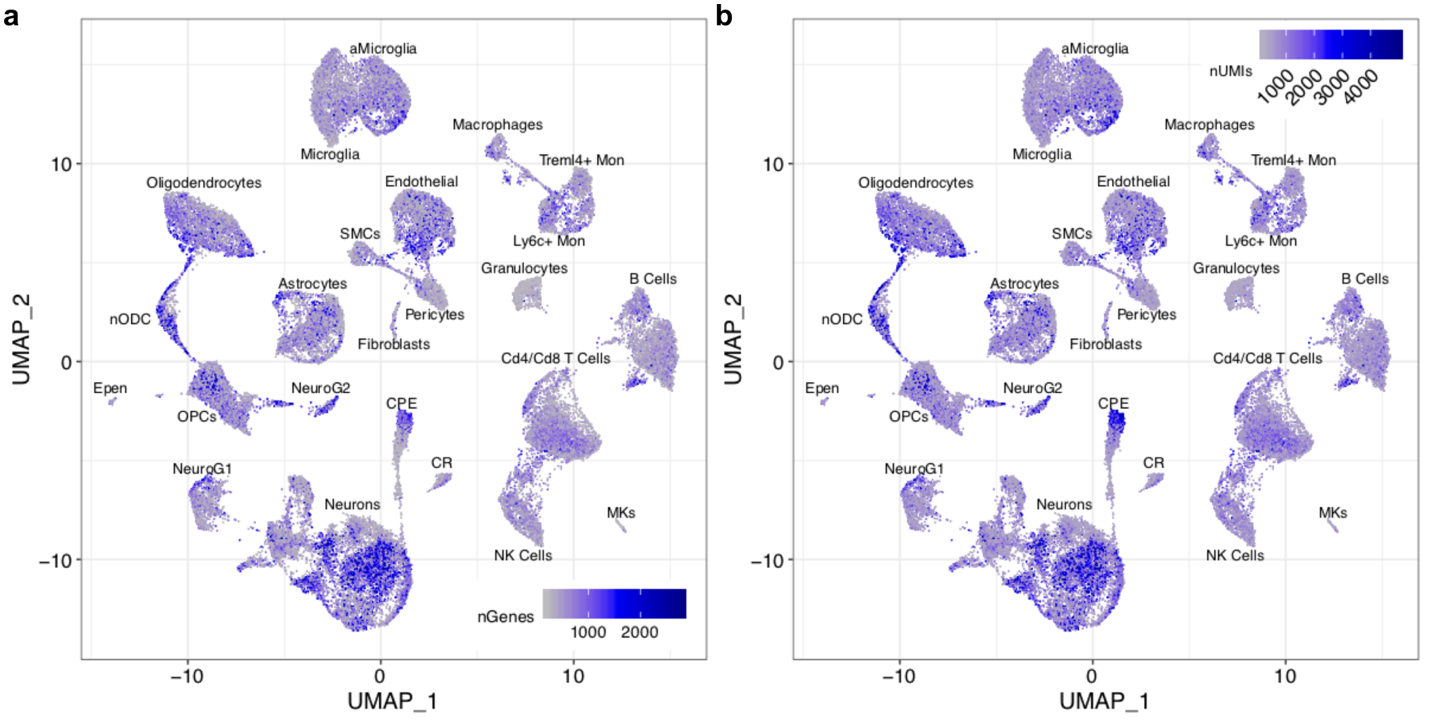
**

**Supplementary Figure 1.** Gene and UMI counts of UMAP embeddings of 78,895 cells from three tissues (frontal cortex, hippocampus, and peripheral blood), two timepoints (24 hours and 7 days), and two conditions (TBI and sham control). Each point represents a single cell. **(a)** UMAP plot with each cell colored by the number of genes expressed by that cell. **(b)** UMAP plot with each cell colored by the number of UMIs counted from that cell.

**Supplementary Figure 2**

**
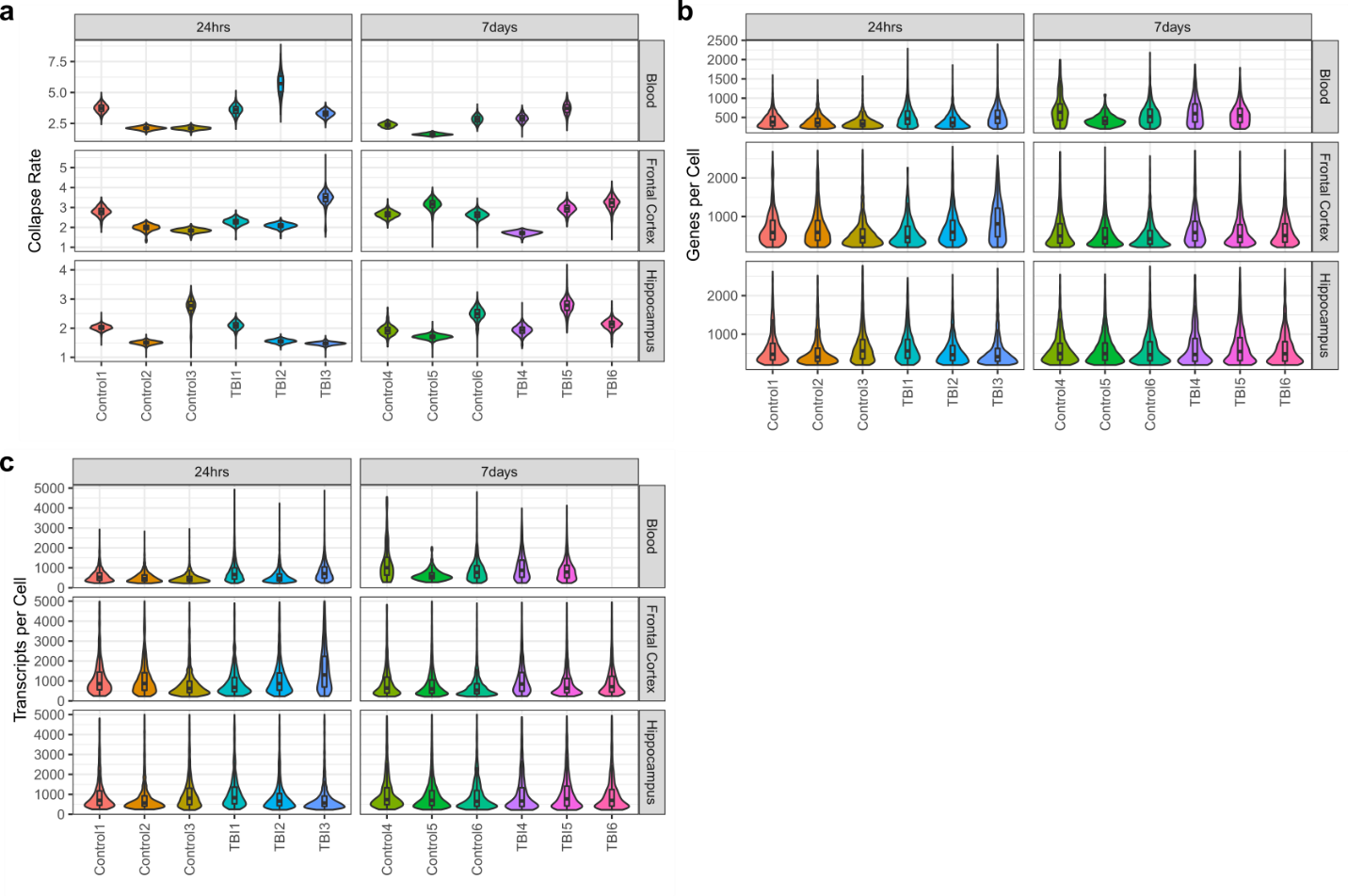
**

**Supplementary Figure 2.** Drop-seq library statistics and quality control (QC) features. **(a-c)** Comparison of QC features of the 35 samples indicate that although the sequencing depth varies somewhat across the samples/libraries, the number of genes and transcripts detected per cell are similar across samples. **(a)** Violin/boxplot for unique molecular identifier (UMI) collapse rate for each sample, calculated as (the total number of UMIs)/(number of unique UMIs) for each single cell in a library. UMI collapse rate is an indicator of how deeply a library is sequenced and how many reads are being discarded. **(b)** Violin/boxplot for the number of unique genes detected per single cell in each sample. **(c)** Violin/boxplot for the number of unique transcripts detected per single cell in each sample. No cells from the 7-day peripheral blood sample TBI6 passed the QC criteria including mitochondrial fraction, the minimum number of transcripts/genes per cell, etc., so this sample could not be analyzed for this tissue/timepoint.

**Supplementary Figure 3**

**
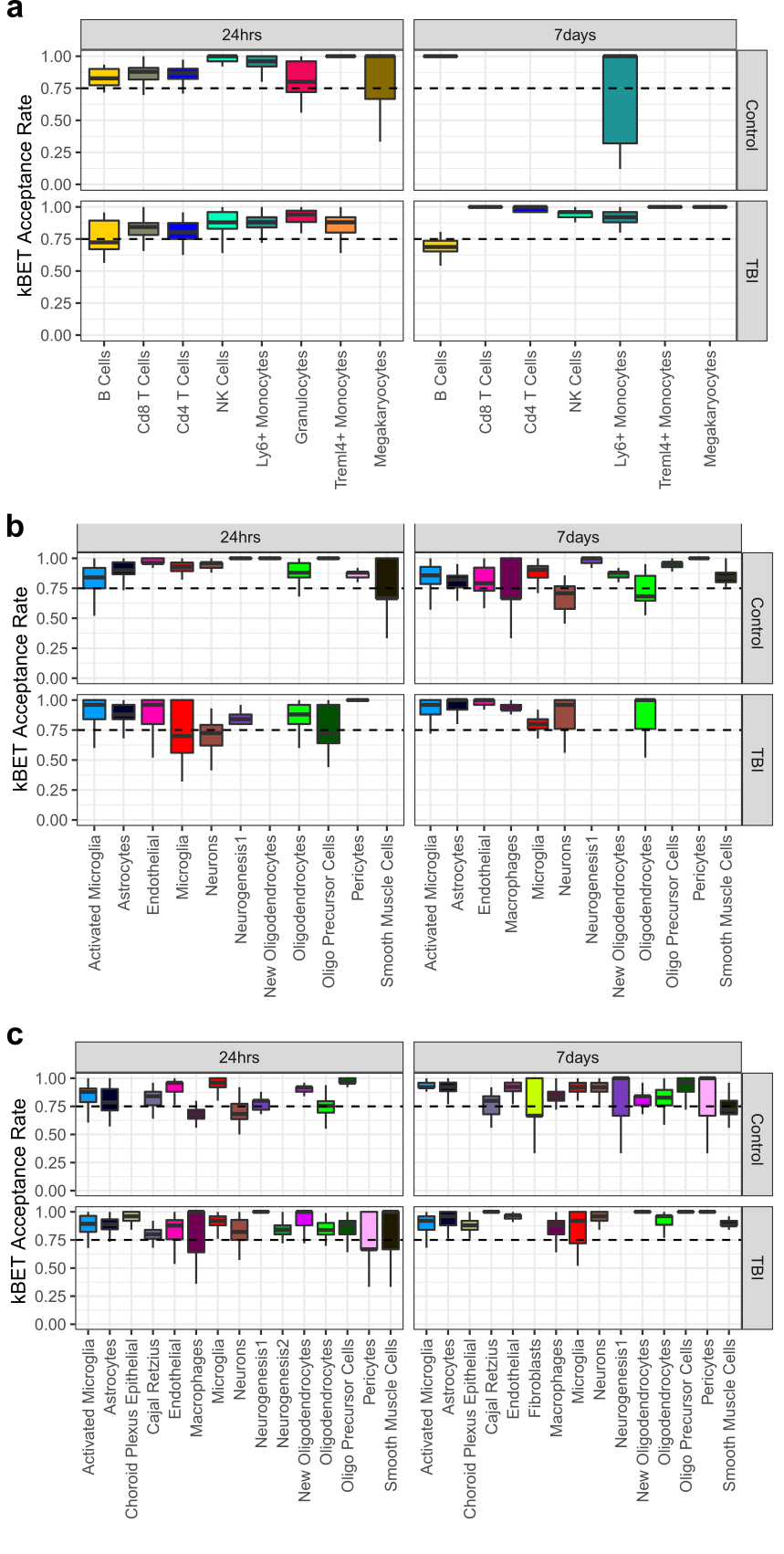
**

**Supplementary Figure 3.** Quantification of batch effect using k-nearest-neighbor batch-effect test (kBET). **(a-c)** kBET was applied on single cells for each cell type which had a sufficient number of single cells from each sample (≥15). kBET was run on samples from the same timepoint (24hrs or 7 days) and condition (TBI or sham control) on **(a)** peripheral blood, **(b)** frontal cortex, **(c)** hippocampus. Boxplots show the median value (line), the 25^th^ and 75^th^ percentiles (edges), and largest values which are 1.5 * interquartile range (whiskers). Dotted line is at 75% kBET acceptance rate which is the value used for well-mixed batches here. All samples for a given cell type within a specific timepoint and condition were required to have ≥15 cells to be considered for the test; missing boxplots did not meet these criteria.

**Supplementary Figure 4**

**
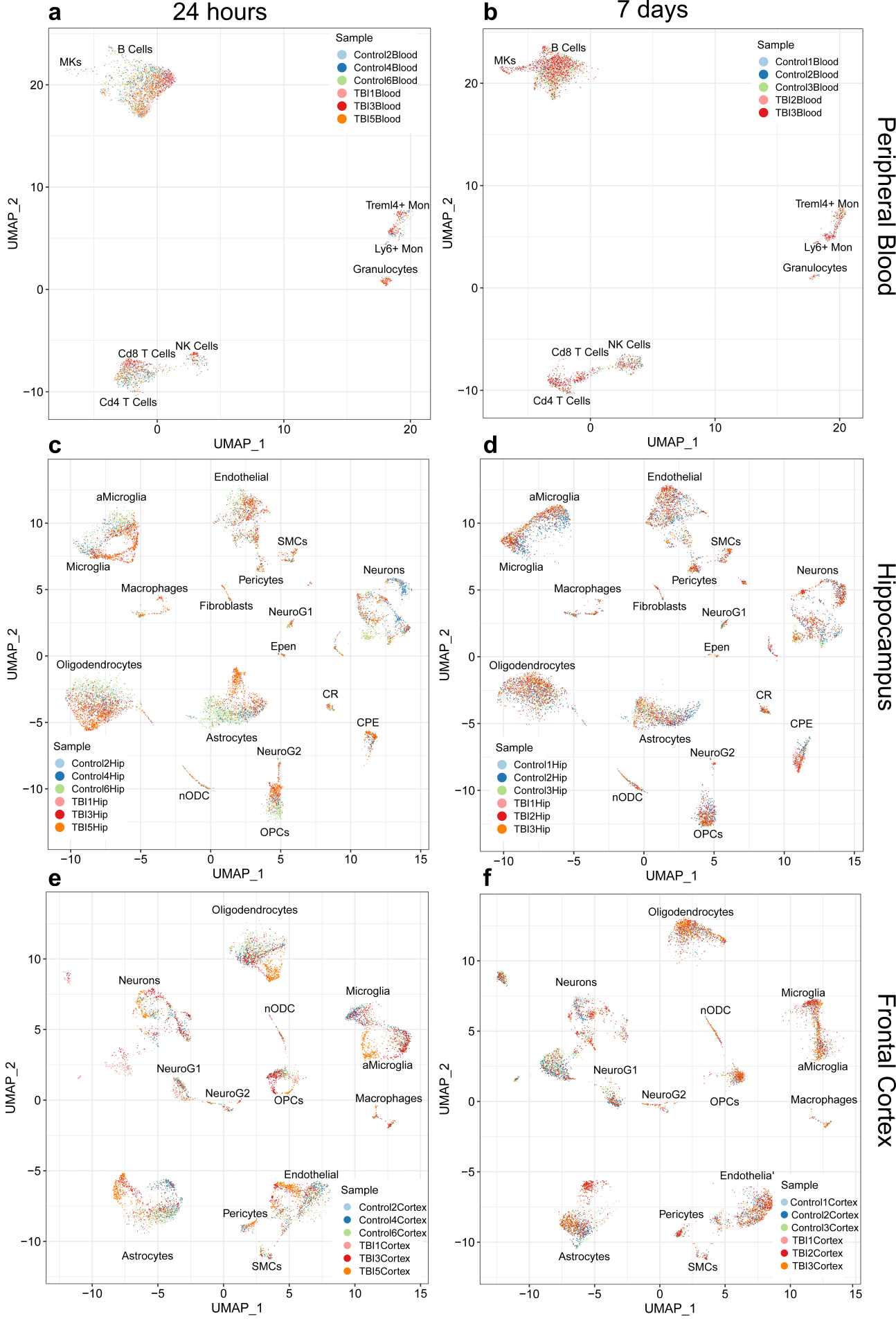
**

**Supplementary Figure 4.** UMAP embeddings of peripheral blood, hippocampus, and frontal cortex single cells colored by sample. **(a-b)** Top panels are samples from peripheral blood, **(c-d)** middle panels are samples from hippocampus, **(e-f)** bottom panels are samples from frontal cortex. **(a,c,e)** Left panels are samples from 24 hours and **(b,d,f)** right panels are samples from 7 days.

**Supplementary Figure 5**

**
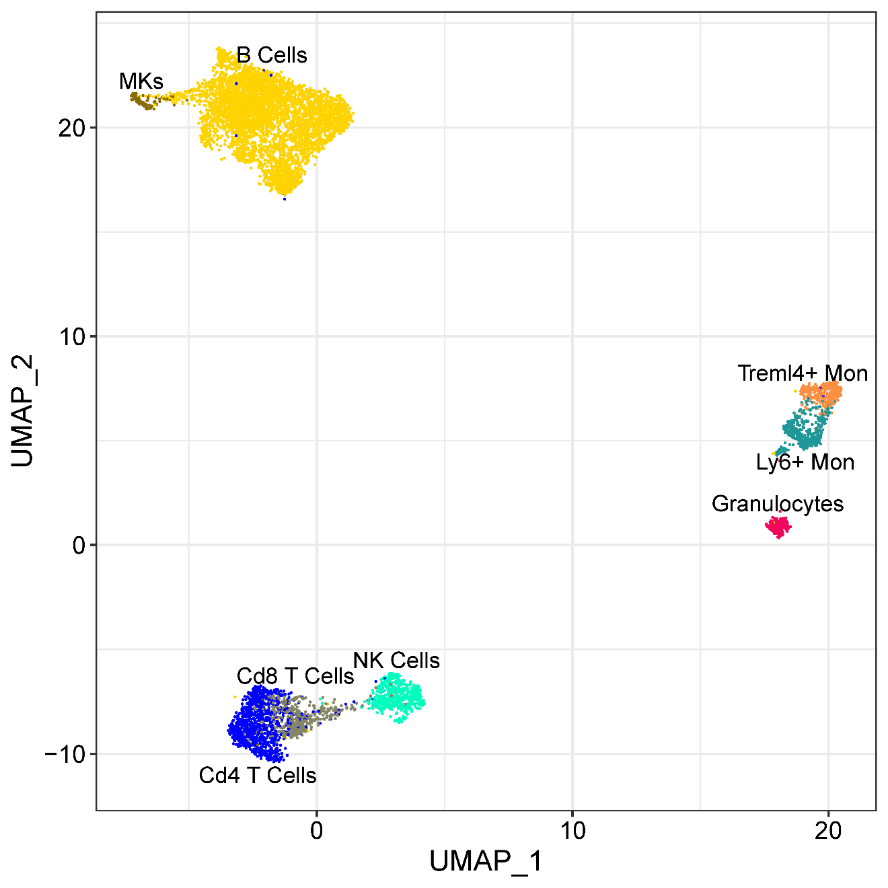
**

**Supplementary Figure 5.** UMAP embeddings of peripheral blood single cells from two timepoints (24 hours and 7 days) and two conditions (TBI and sham control). UMAP plot with each cell colored according to the cell type identity.

**Supplementary Figure 6**

**
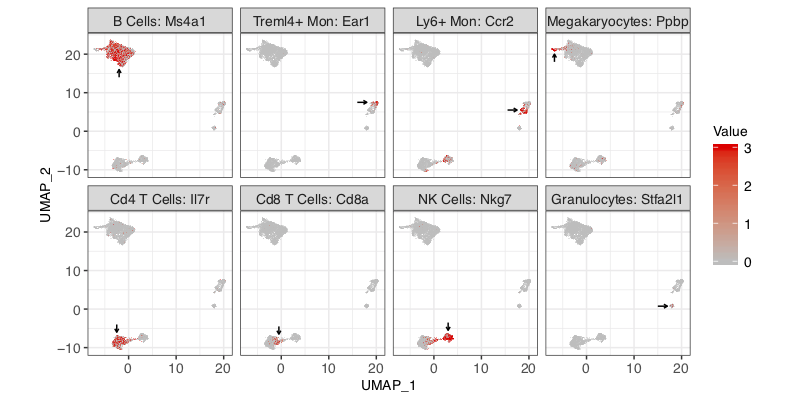
**

**Supplementary Figure 6.** Cluster specific expression of known peripheral blood cell-type specific marker genes. The normalized expression levels of known cell-type specific markers are indicated with red color in UMAP plots to assign cluster identities. The cluster of interest is indicated by the black arrow.

**Supplementary Figure 7**

**
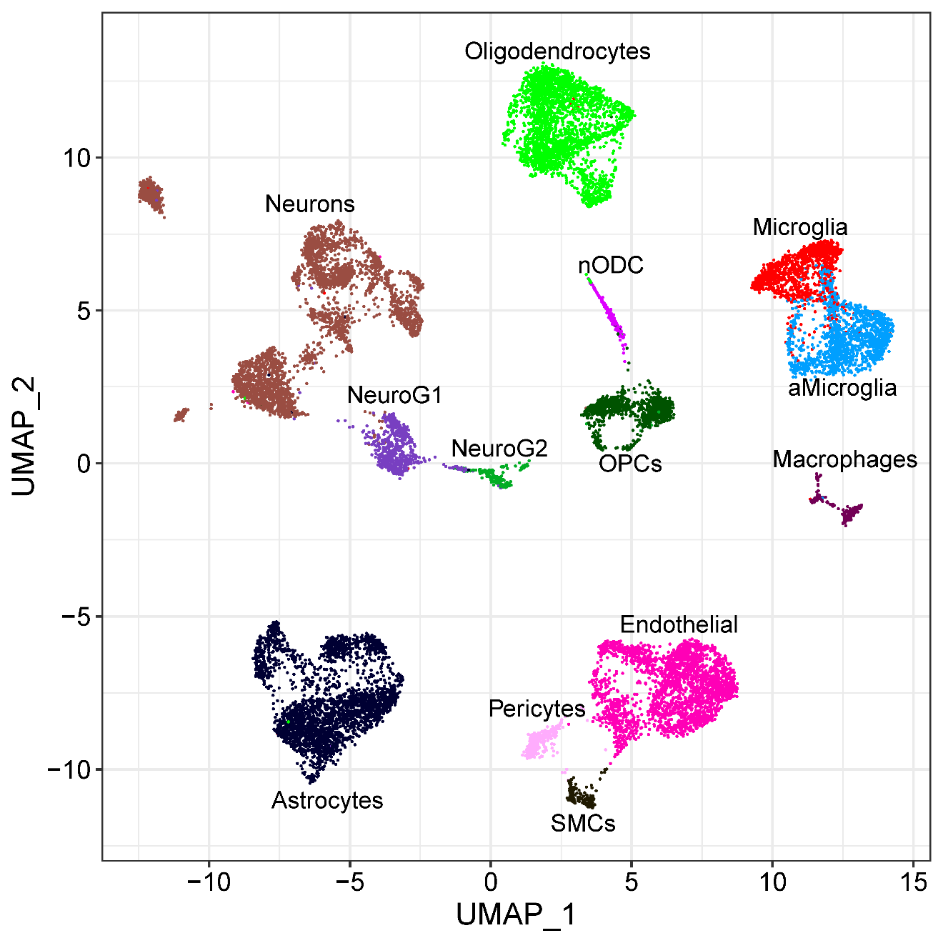
**

**Supplementary Figure 7.** UMAP embeddings of frontal cortex single cells from two timepoints (24 hours and 7 days) and two conditions (TBI and sham control). UMAP plot with each cell colored according to the cell type identity.

**Supplementary Figure 8
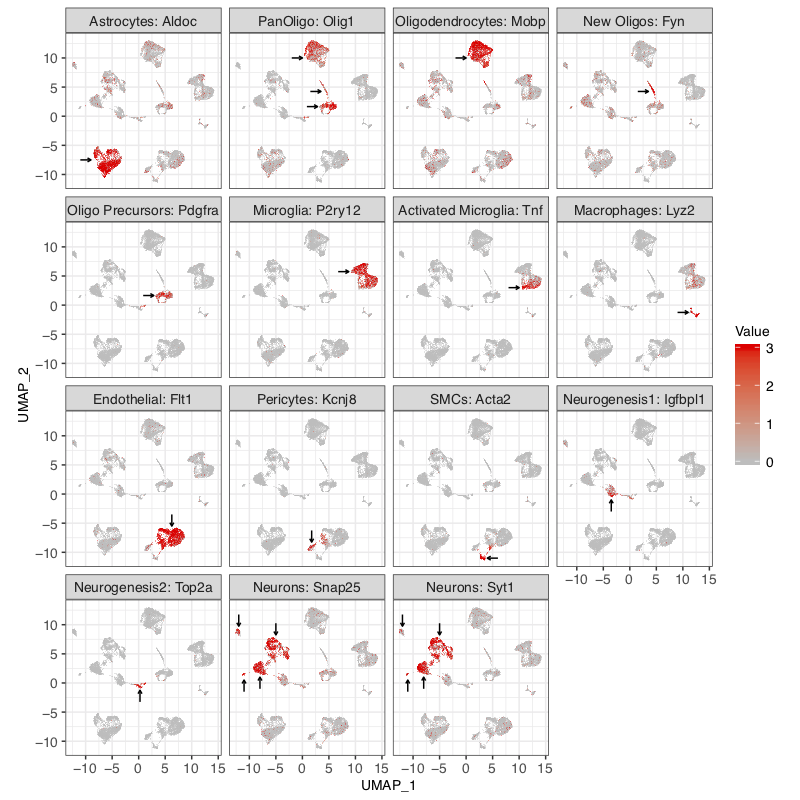
**

**Supplementary Figure 8.** Cluster specific expression of known frontal cortex cell-type specific marker genes. The normalized expression levels of known cell-type specific markers are indicated with red color in UMAP plots to assign cluster identities. The cluster of interest is indicated by the black arrow.

**Supplementary Figure 9**

**
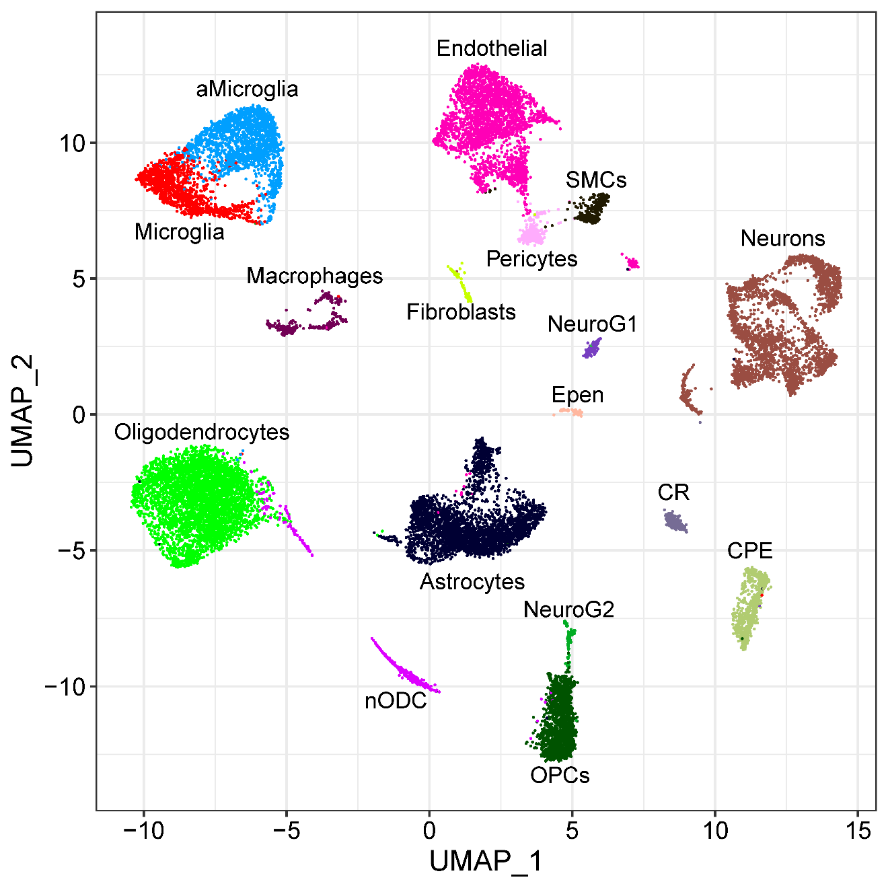
**

**Supplementary Figure 9.** UMAP embeddings of hippocampus single cells from two timepoints (24 hours and 7 days) and two conditions (TBI and sham control). UMAP plot with each cell colored according to the cell type identity.

**Supplementary Figure 10**

**
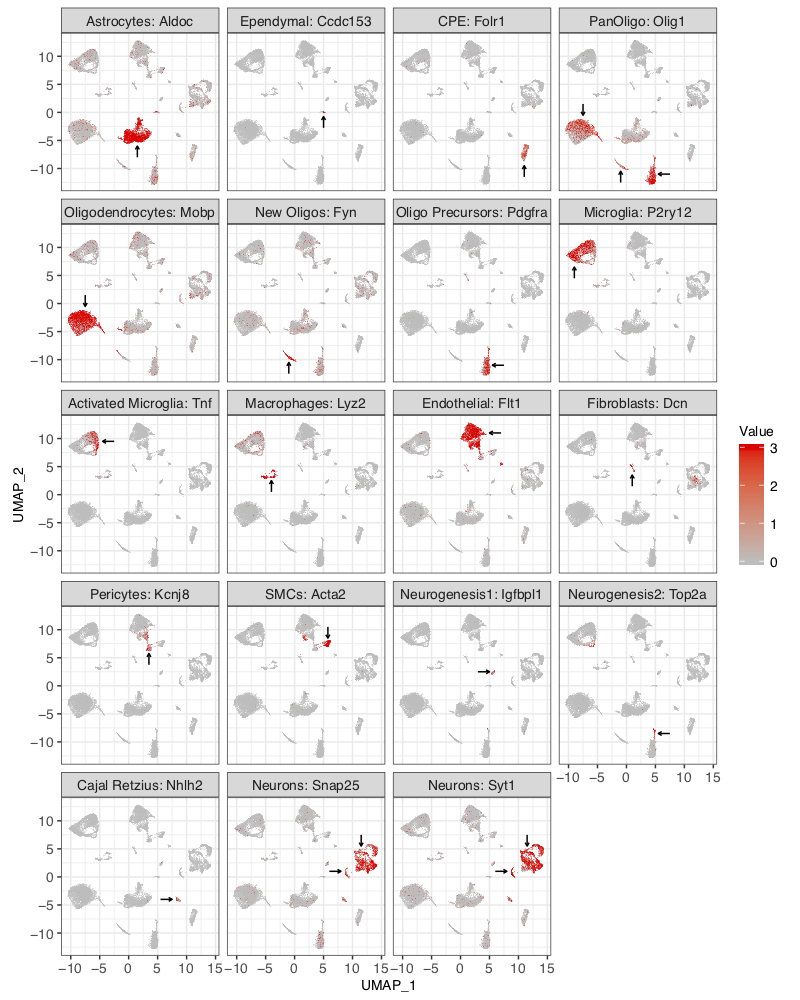
**

**Supplementary Figure 10.** Cluster specific expression of known hippocampus cell-type specific marker genes. The normalized expression levels of known cell-type specific markers are indicated with red color in UMAP plots to assign cluster identities. The cluster of interest is indicated by the black arrow.

**Supplementary Figure 11**

**
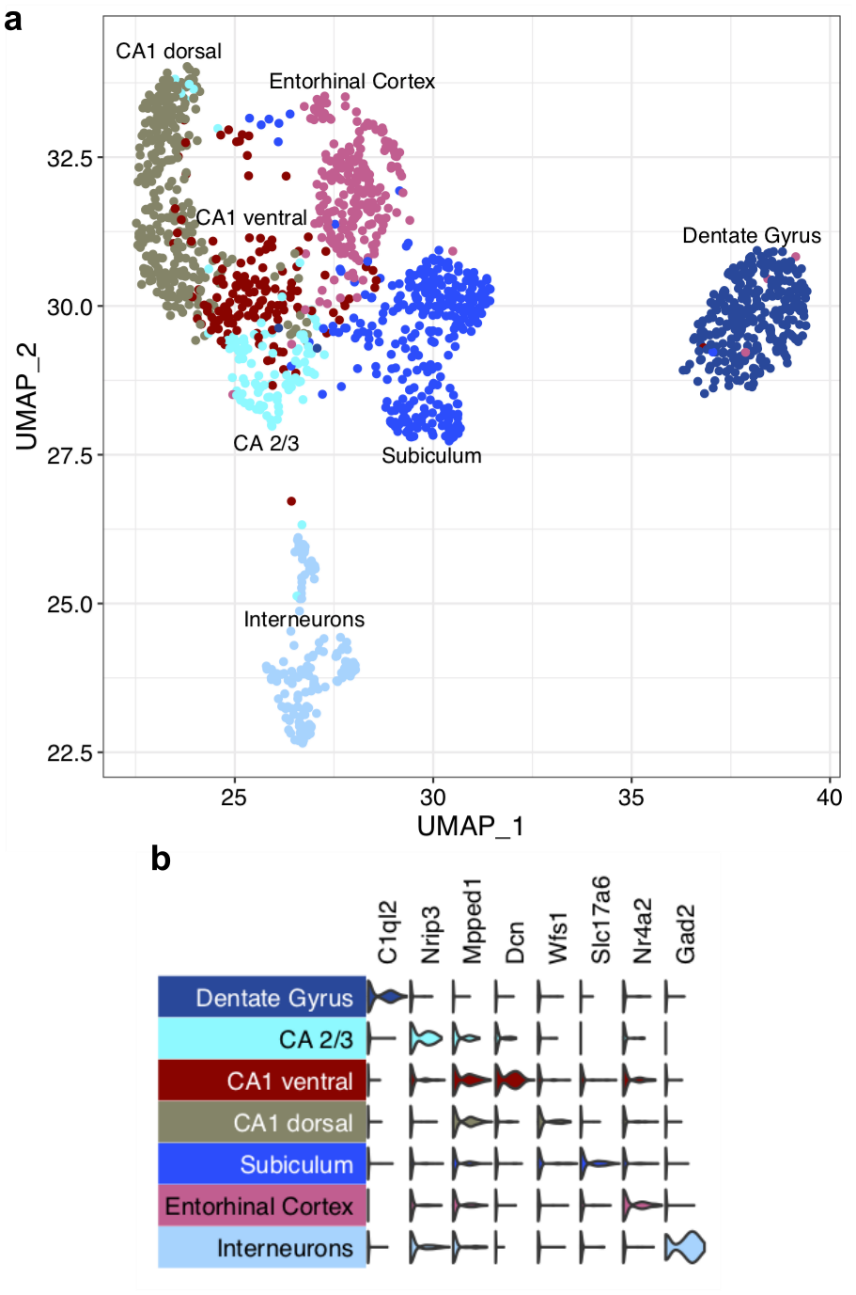
**

**Supplementary Figure 11.** UMAP embeddings of hippocampus neuron single cells from two timepoints (24 hours and 7 days) and two conditions (TBI and sham control). **(a)** UMAP plot with each cell colored according to the cell type identity. **(b)** Normalized expression value of canonical hippocampus neuron cell type marker genes.

**Supplementary Figure 12**

**
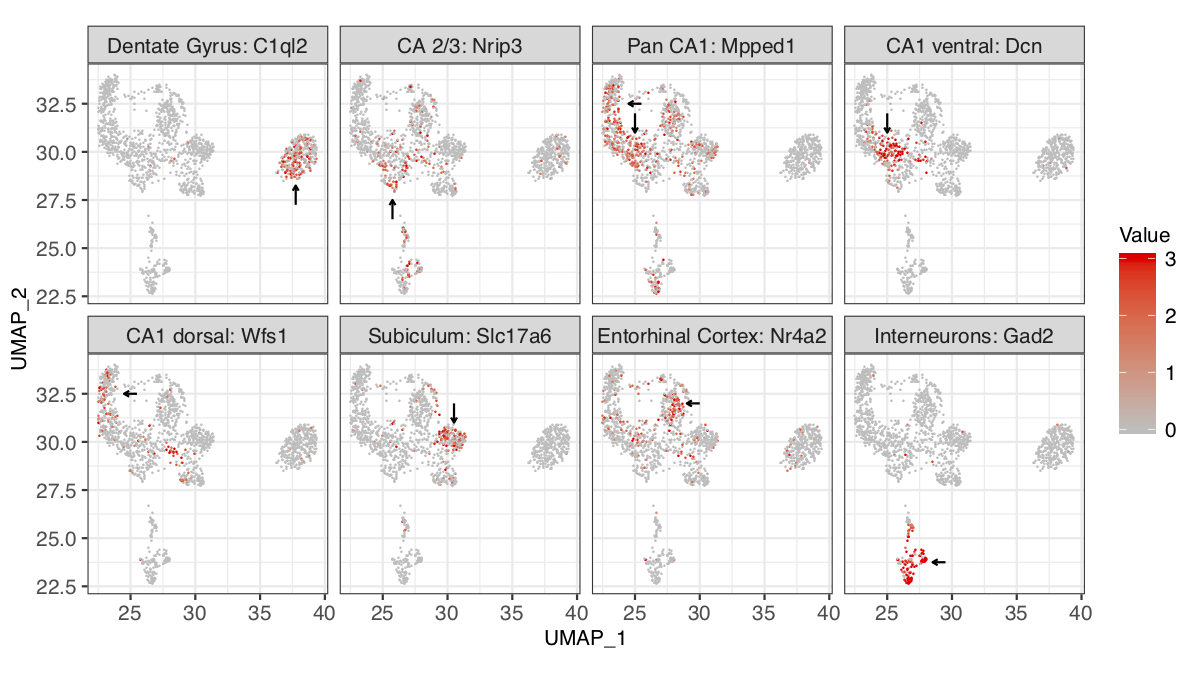
**

**Supplementary Figure 12.** Cluster specific expression of known cell-type specific marker genes of hippocampus neuronal subpopulations. The normalized expression levels of known cell-type specific markers are indicated with red color in UMAP plots to assign cluster identities. The cluster of interest is indicated by the black arrow.

**Supplementary Figure 13**

**
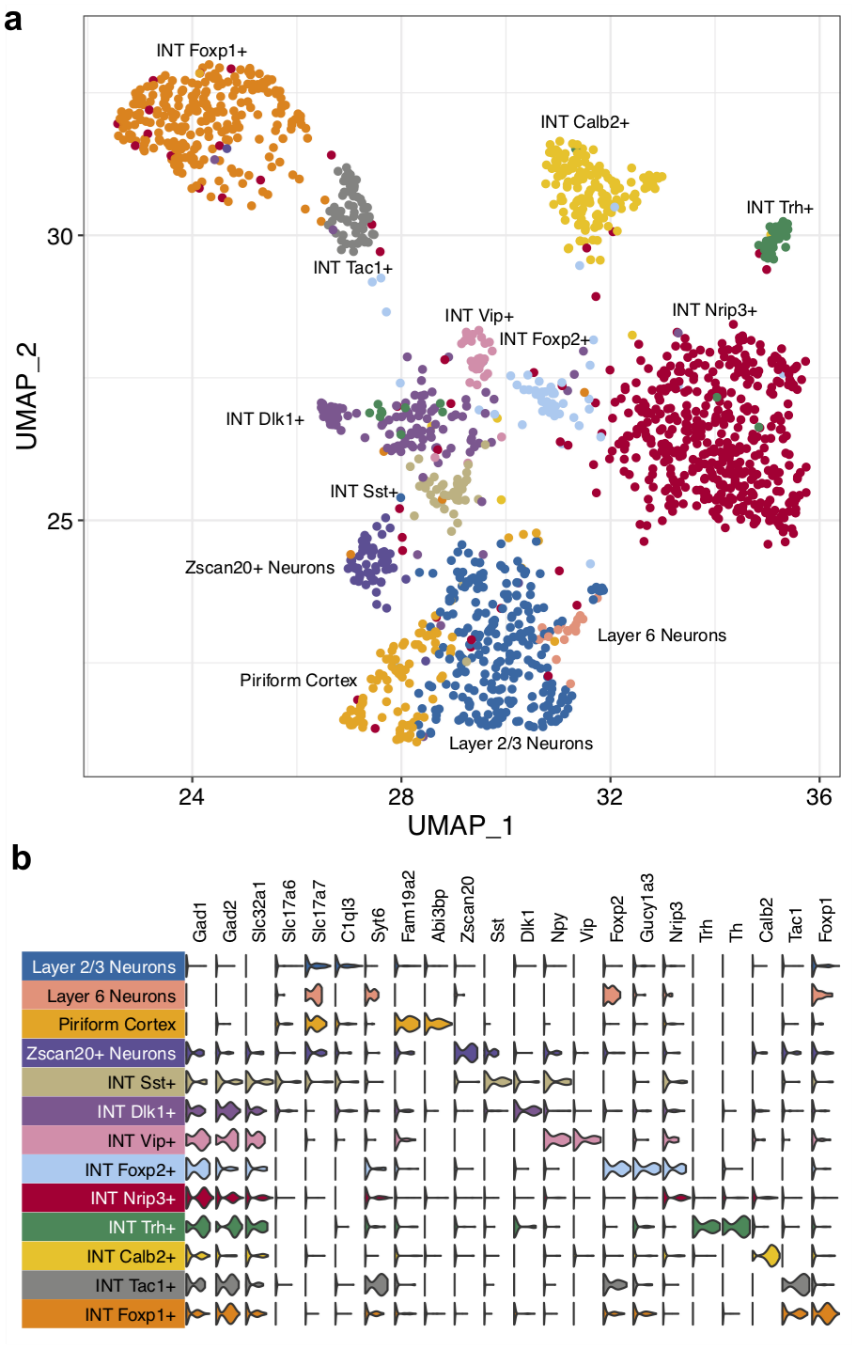
**

**Supplementary Figure 13.** UMAP embeddings of frontal cortex neuron single cells from two timepoints (24 hours and 7 days) and two conditions (TBI and sham control). **(a)** UMAP plot with each cell colored according to the cell type identity. **(b)** Normalized expression value of canonical frontal cortex cell type marker genes.

**Supplementary Figure 14**

**
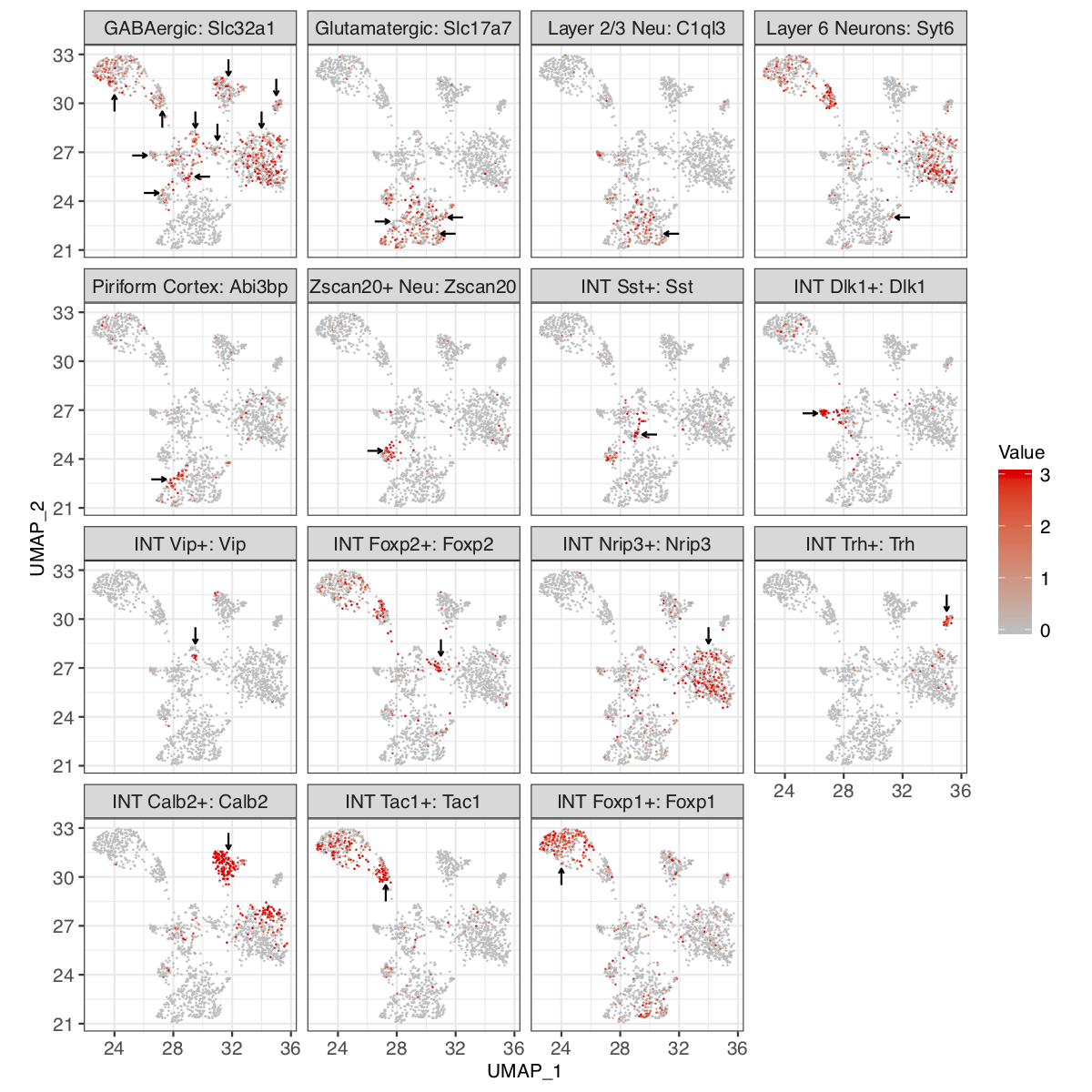
**

**Supplementary Figure 14.** Cluster specific expression of known cell-type specific marker genes of frontal cortex neuronal subpopulations. The normalized expression levels of known cell-type specific markers are indicated with red color in UMAP plots to assign cluster identities. The cluster of interest is indicated by the black arrow.

**Supplementary Figure 15**

**
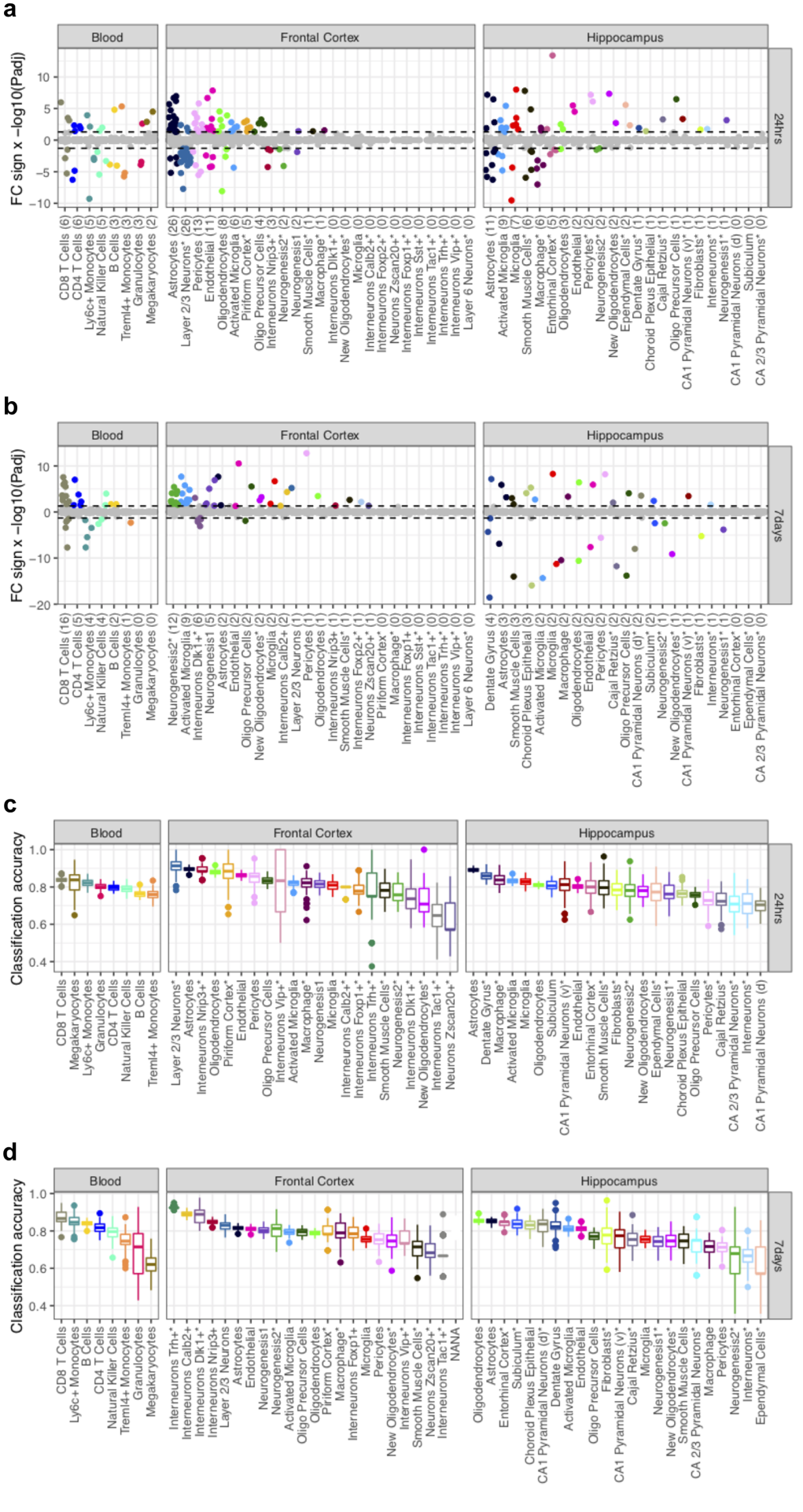
**

**Supplementary Figure 15.** Quantifying transcriptome-wide effect of TBI on individual cell populations. **(a-b)** Number of DEGs between TBI and sham control cells within the same cell type. To make the power comparable across cell types, each cell type has been subsampled to 100 cells and cell types with a “*” possessed less than 100 cells. The number of significant DEGs (adjusted p-value < 0.05) is indicated in parenthesis. Dotted lines indicate significance and colored points are significant DEGs. The y-axis is the -log10 of the adjusted p-value with the sign determined by the sign of the fold change (e.g. negative values have a negative fold change). Panels are split by timepoint: **(a)** 24 hours and **(b)** 7 days. Cell types in each panel are ranked based on the numbers of DEGs, i.e., numbers in the parenthesis, detected. **(c-d)** Classification accuracy of SVM classifier to predict sham control or TBI labels within each cell type based on the top 1000 most highly expressed genes for that cell type at **(c)** 24 hours and **(d)** 7 days.

**Supplementary Figure 16**

**
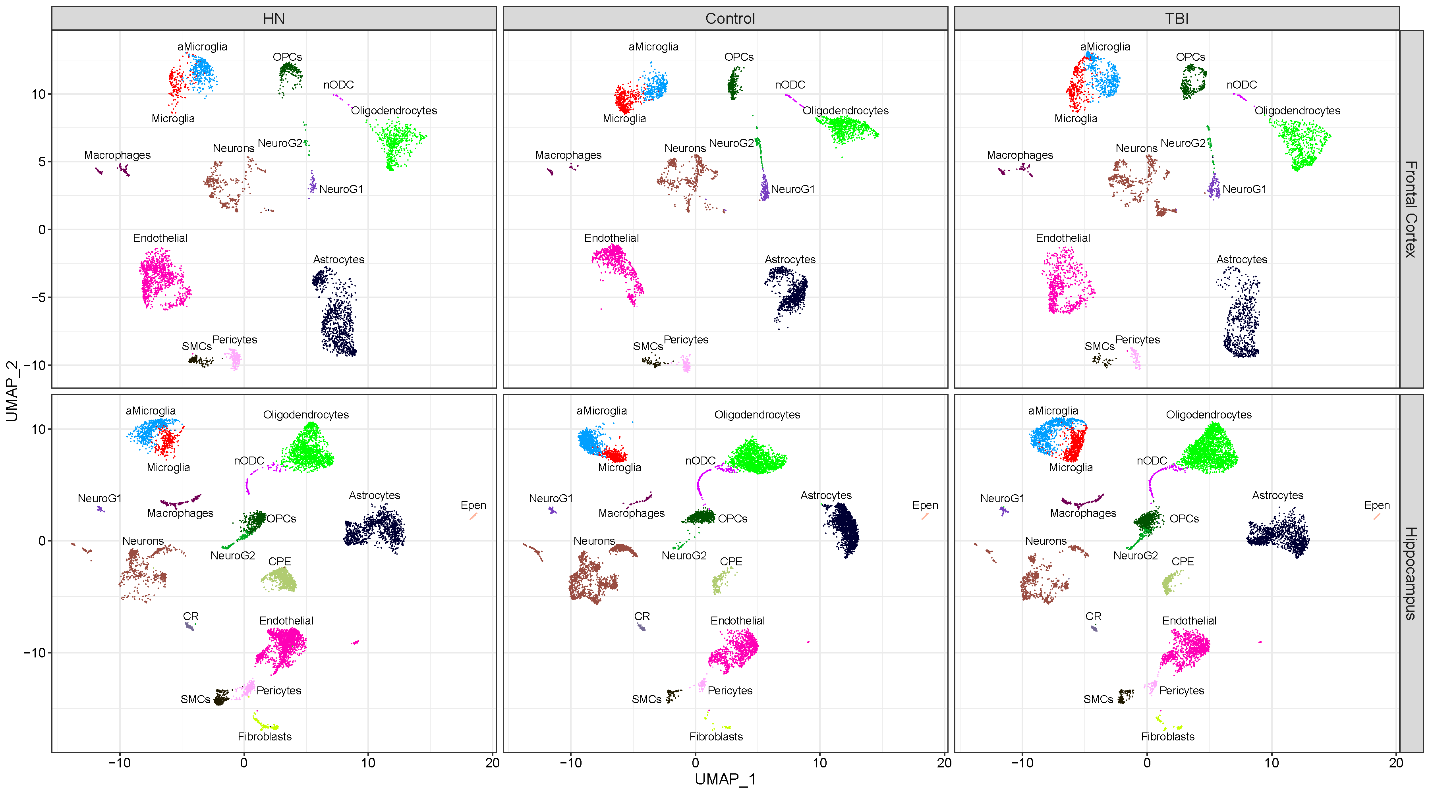
**

**Supplementary Figure 16.** UMAP embeddings of frontal cortex and hippocampus single cells from three conditions (humanin-treated TBI, sham control, and TBI) at 24 hours post-TBI. UMAP plot with each cell colored according to the cell type identity.
